# Quantification of Protein Glycosylation Using Nanopores

**DOI:** 10.1021/acs.nanolett.2c01338

**Published:** 2022-06-29

**Authors:** Roderick Corstiaan Abraham Versloot, Florian Leonardus Rudolfus Lucas, Liubov Yakovlieva, Matthijs Jonathan Tadema, Yurui Zhang, Thomas M. Wood, Nathaniel I. Martin, Siewert J. Marrink, Marthe T. C. Walvoort, Giovanni Maglia

**Affiliations:** † Groningen Biomolecular Sciences and Biotechnology Institute, University of Groningen, 9747AG Groningen, The Netherlands; ‡ Chemical Biology Division, Stratingh Institute for Chemistry, University of Groningen, 9747AG Groningen, The Netherlands; § Biological Chemistry Group, Institute of Biology Leiden, Leiden University, 2333 BE Leiden, The Netherlands

**Keywords:** protein glycosylation, single molecule, nanopore spectrometry, rhamnosylation, proteomics

## Abstract

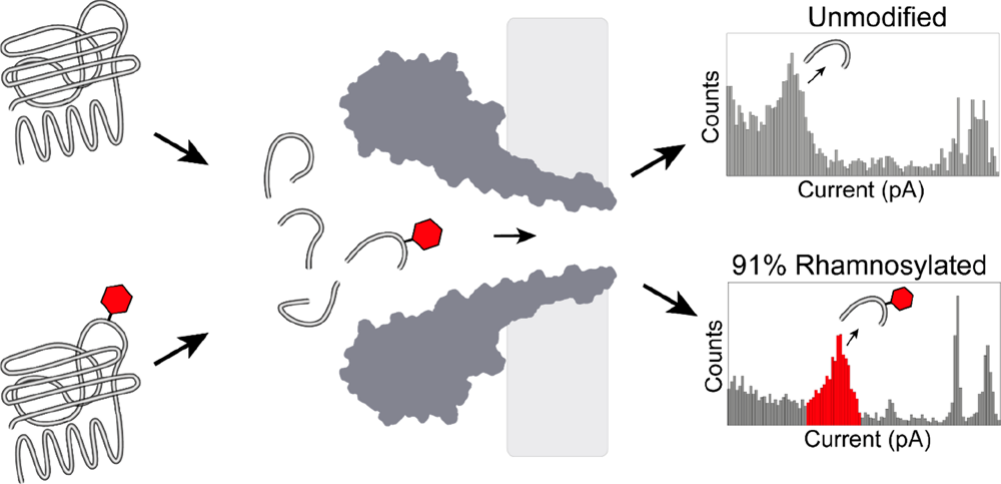

Although nanopores can be used for single-molecule sequencing of nucleic acids using low-cost portable devices, the characterization of proteins and their modifications has yet to be established. Here, we show that hydrophilic or glycosylated peptides translocate too quickly across FraC nanopores to be recognized. However, high ionic strengths (i.e., 3 M LiCl) and low pH (i.e., pH 3) together with using a nanopore with a phenylalanine at its constriction allows the recognition of hydrophilic peptides, and to distinguish between mono- and diglycosylated peptides. Using these conditions, we devise a nanopore method to detect, characterize, and quantify post-translational modifications in generic proteins, which is one of the pressing challenges in proteomic analysis.

One of the main challenges in proteomics is the identification of protein isoforms and especially post-translational modifications (PTMs), among which protein glycosylation is one of the most abundant modifications in nature.^1^ Protein glycosylation is often detected using affinity-based approaches based on saccharide-binding proteins such as lectins^2,3^ and antibodies,^4^ through the attachment of fluorescent probes,^5,6^ or via mass spectrometry-based approaches.^7^ Although affinity-based detection can identify glycosylated residues with high specificity, only a limited range of proteins or glycans can be targeted. Mass spectrometry (MS), on the other hand, is a powerful technique for the global analysis of glycoproteins in complex samples.^8−10^ However, low-abundance proteins are notoriously difficult to detect using MS approaches, as their signal is clouded by other proteins.^11^ In addition, MS facilities often require sophisticated devices that are expensive to maintain and run, and technical complications arise from detecting substochiometric PTMs, where only a fraction of the proteins are modified.^12^

Biological nanopores are a class of promising single-molecule biosensors that can be integrated into low-cost, high-throughput, and portable devices that have been adapted to the detection and analysis of proteins^13−17^ and peptides.^18−22^ In a typical experiment, an external bias is applied across a single nanopore embedded into a nonconducting amphipathic membrane separating two electrolyte solutions. When an analyte passes through the nanopore, the ionic flow of the open pore is transiently blocked. The excluded current (*I*_ex_% = (*I*_B_*–I*_O_)/*I*_O_ × 100%), the ratio between the current blockade (*I*_O_ – *I*_B_) and open pore current (*I*_O_) has been shown to mainly depend on the volume of the analyte,^19,23−26^ although the physicochemical properties of the analyte as well as its interactions with the nanopore surface and buffer components can also play a part.^27−30^

A few reports have claimed the identification of PTMs in peptides and proteins. Using an immobilized polypeptide^17^ or an electrophoretically trapped peptide,^31^ it was reported that differences between glycosylation and phosphorylation could be measured. Later, differences in the ionic signal due to the acetylation or phosphorylation of a tau peptide were also observed.^32,33^ However, these reports limited their analysis to bespoke (poly)peptides and circumvented the critical issue of the fast translocation of natural peptides and their modification generated from the proteolytic cleavage of proteins. Therefore, the investigation of PTMs in proteins could not be addressed. Earlier attempts to investigate proteins bearing a PTM were also made. We and others showed that unmodified and ubiquitinated proteins have a different signal,^34−36^ which could be used to follow the ubiquitination reaction.^34^ It was also reported that nanopores functionalized with an antibody can distinguish between its cognate glycosylated or unglycosylated protein.^16^ However, since the relationship between the current signal and the modification depended on the specific properties of each individual system, these studies did not provide a method for the detection and quantification of PTMs in generic proteins.

Here, we present the successful identification of glycopeptides from natural proteins. We found that hydrophilic (glyco-) peptides translocated too quickly through nanopores to be observed under a range of conditions. However, by combining an aromatic sensing region within the nanopore together with high electrolyte concentrations, we revealed that glycopeptides were efficiently detected at low pH. Using these conditions, we detected and quantified rhamnosylation of cyclic peptides and proteins. Therefore, this work shows that nanopores can be used similarly to mass spectrometry for the detection and quantification of PTMs in proteins.

## Detection of Glycopeptides Using FraC

**Figure 1:**
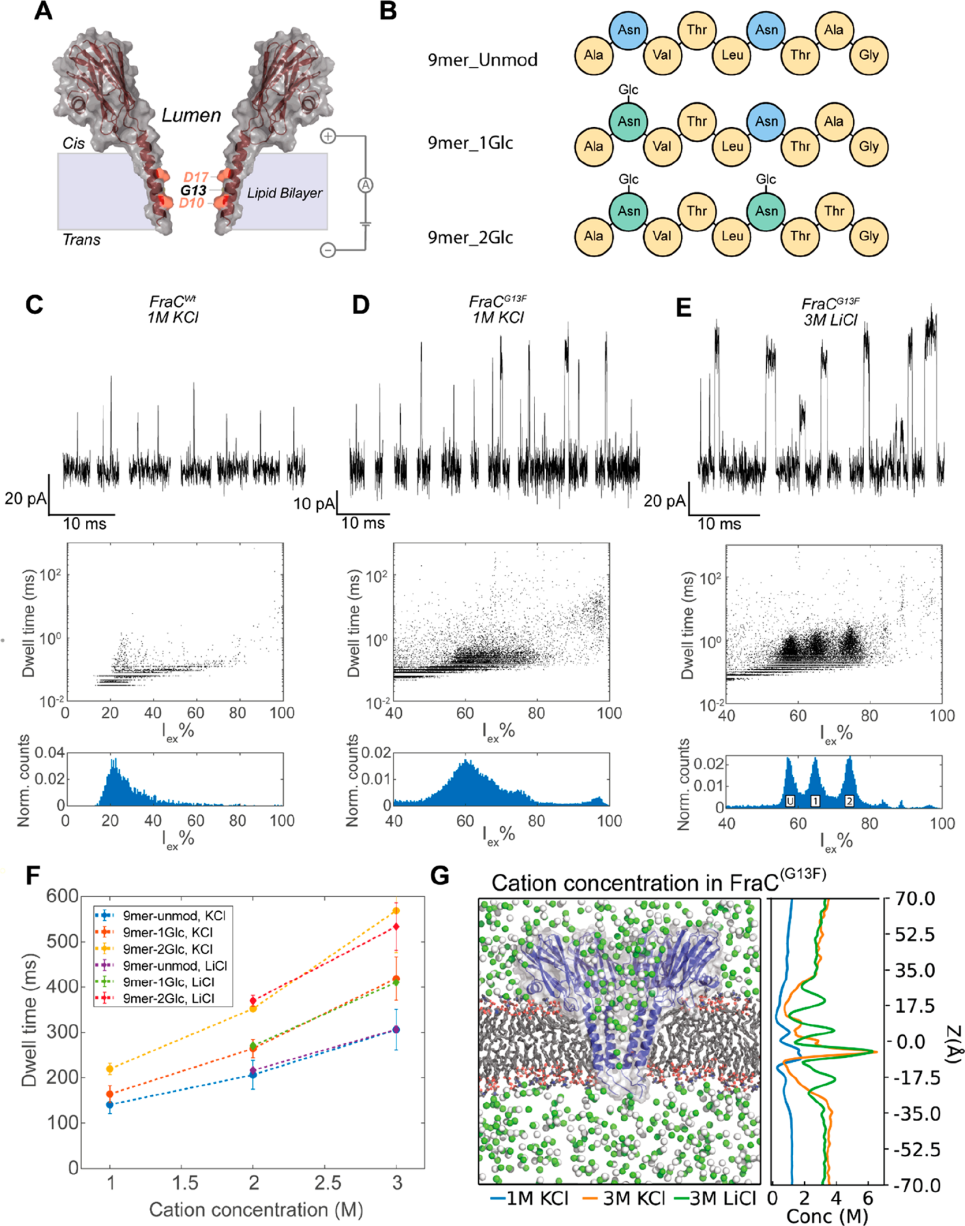
Detection of glycopeptides in FraC nanopores. (A) Schematic representation of a FraC monomer. The lumen-facing residues in the constriction of the pore are indicated. (B) Schematic representation of the composition of the peptides used. (C–E) Representative events (top), dwell time versus excluded current (middle) and excluded current histogram (bottom) of an equimolar mixture of 9mer_unmod, 9mer_1Glc, and 9mer_2Glc measured in (C) FraC^Wt^ in 1 M KCl and 10 μM peptide mixture, (D) FraC^G13F^ in 1 M KCl and 2.5 μM peptide mixture, (E) FraC^G13F^ in 3 M LiCl and 5 μM peptide mixture. The location of peaks in the histogram belonging to 9mer_unmod (U), 9mer_1Glc (1), and 9mer_2Glc (2) are indicated. Data were recorded at 50 kHz sampling frequency, with a 10 kHz Bessel filter at pH 3.8. (F) Dwell time of the glycopeptides in buffers with varying salt concentrations. (G) The left panel shows a cut through of a MD simulation of a FraC^G13F^ nanopore (blue) in a lipid bilayer (gray) in the presence of 3 M concentration of potassium (green) and chloride (white) ions at pH 3.8. The right panel shows the cation concentration along the *z*-axis averaged over 20 ns of MD simulation trajectory under a −50 mV potential.

In planar lipid bilayers FraC nanopores assemble in three possible oligomeric forms: named type I (octamer), type II (heptamer) or type III (hexamer).^37^ Previous work indicated that a variety of generic peptides can be analyzed using FraC nanopores in 1 M KCl at pH 3.8.^19,20,37,38^ However, we found that under the same conditions, FraC^Wt^ nanopores (Figure 1A), or a range of variants (FraC^G13F^, FraC^D10R^, FraC^G13W^, FraC^G13H^, all containing a mutation in the constriction of the pore that was previously shown to enhance peptide recognition),^20^ cannot accurately detect neutral and hydrophilic model peptides of nine residues containing zero (9mer_unmod, ANVTLNTAG), one (9mer_1Glc, ANVTLNTAG, glycosylation site underlined), or two (9mer_2Glc, ANVTLNTTG) glucose (Glc) modifications (Figure 1B, Figures S1–S3). Fast translocation events were observed, but the peptides translocated too quickly to accurately determine their *I*_ex_% (Figure 1C,D, Figures S4 and S5). Nanopore variant FraC^G13F^ was able to detect more peptides than FraC^Wt^ nanopores; the event frequency increased from 0.03 ± 0.02 s^–1^ μM^–1^ for FraC^Wt^ to 3.9 ± 0.9 s^–1^ μM^–1^ for FraC^G13F^ (Table S1). The increased detection most likely originates from interactions of the phenylalanine with positively charged peptides (via cation−π interactions) and with carbohydrates (via π-stacking interactions).^39^ Both interactions are expected to increase the dwell time of glycopeptides in the nanopore. The resolution, however, was still insufficient to distinguish between glycosylated and nonglycosylated peptides (Figure 1D). It has been shown previously that the constriction region of FraC near D10 (Figure 1A) contains an electrostatic barrier, formed by the congregation of potassium ions.^20^ We reasoned that by increasing the salt concentration or by using cations with a smaller diameter, more cations will congregate around the sampling region of FraC, thereby tuning the electrostatic barrier in the pore, allowing a stronger interaction between the peptide and the nanopore.^40^ In addition, high ionic strengths can reduce the electroosmotic flow across a nanopore,^23^ which in turn may reduce the translocation speed. We tested different concentrations of KCl and LiCl buffers and found that in 3 M LiCl solution, the three peptides were identified as separate clusters, with little overlap between the peaks in the residual current histogram (Figure 1E). When we compared the dwell time of glycopeptides in different buffers, we observed an increase of the average dwell time when a higher salt concentration was used, but there was little difference between LiCl and KCl buffers of the same concentration (Figure 1F). MD simulations of FraC^G13F^ in different salt concentrations (1 M KCl, 3 M KCl and 3 M LiCl) at pH 3.8 revealed that the concentration of potassium ions around residue D10 is much higher in 3 M KCl solution compared to 1 M KCl (Figure 1G), whereas the peak concentration in 3 M KCl and 3 M LiCl is similar. Therefore, most likely, the increased congregation of cations in the constriction of FraC^G13F^ in high-salt solutions increased the dwell time and allowed more accurate measurements of glycopeptides.

We characterized nanopore events in 3 M LiCl buffer using the relative excluded current and the dwell time. As expected from a signal originating from the excluded volume of peptides, the nonmodified peptide had a lower excluded current [*I*_ex_% = (*I*_B_ – *I*_O_)/*I*_O_ × 100% = 57.9 ± 0.1%], which increased for the monoglycosylated peptide (64.9 ± 0.1%) and diglycosylated peptide (74.2 ± 0.1%). We previously showed using synthetic peptides that mimic trypsinated lysozyme in FraC^G13F^ nanopores in 1 M KCl that, since the excluded current is directly proportional to the volume of the peptide, the mass vs excluded current relation can be reasonably described by a second-order polynomial:
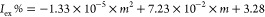
1where *m* is the peptide mass expressed in Da.^20^ The *I*_ex_% estimated from eq 1 followed the same trend as the measured *I*_ex_%, where the *I*_ex_% increases by approximately 8% upon glycosylation (55.6% for the unmodified peptide, 63.3% for the monoglycosylated peptide, and 71.5% for the diglycosylated peptide). The small difference between the estimated *I*_ex_% and measured *I*_ex_% is likely due to the higher ionic strength and different cation used in the nanopore measurements here.

## Quantification of Rhamnosylation on Cyclic Peptides

**Figure 2:**
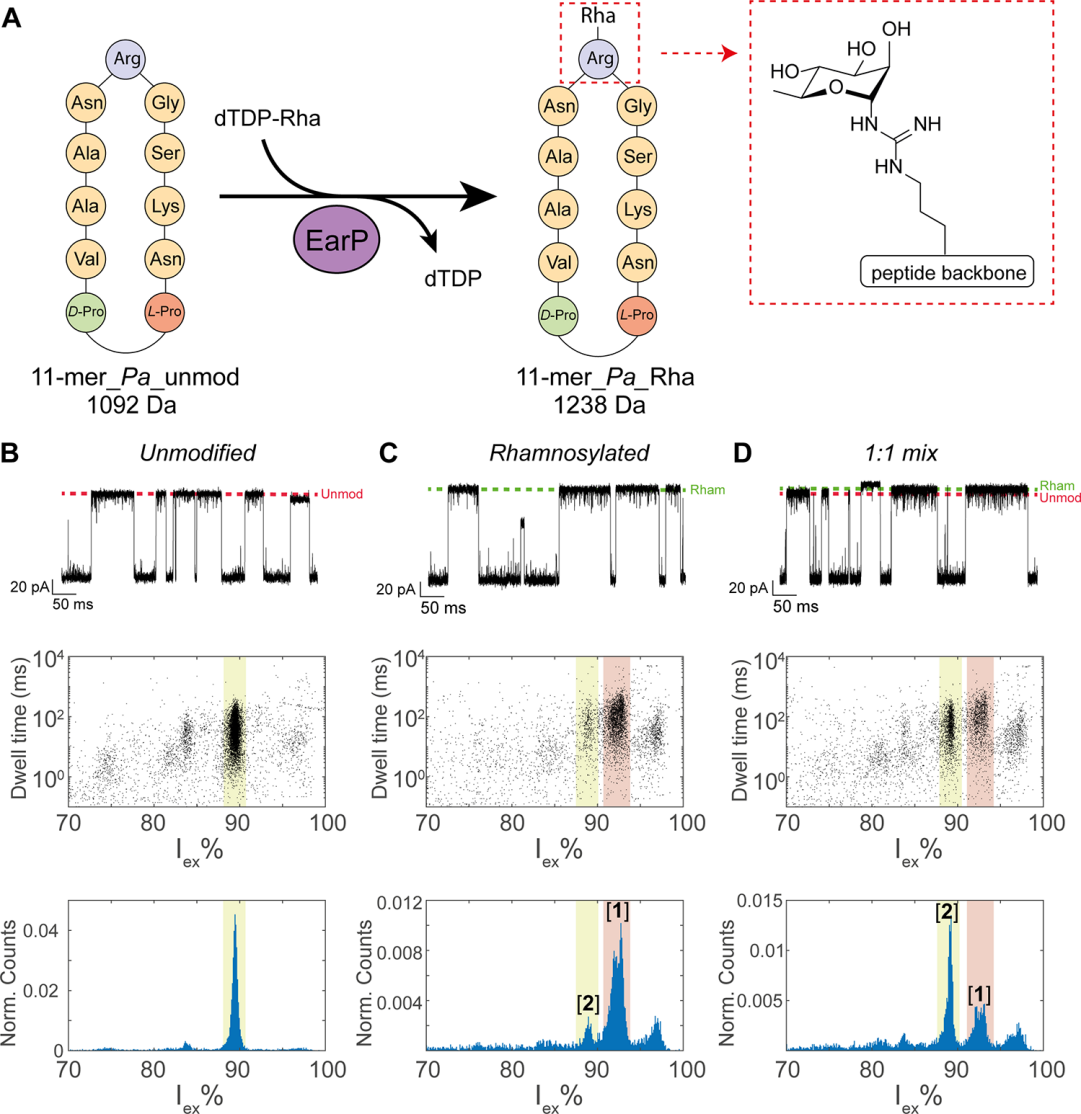
Nanopore detection of rhamnosylation in cyclic peptides. (A) Schematic representation of the cyclic peptides before and after the rhamnosylation reaction. (B–D) Characteristic events (top), dwell time versus excluded current (middle), and excluded current histogram (bottom) of (B) 2.5 μM 11-mer_*Pa*_unmod, (C) 2.5 μM 11-mer_*Pa*_Rha, and (D) their 1:1 mixture in a final concentration of 2.5 μM. The dotted lines indicate the approximate ionic current levels of the unmodified (red) and rhamnosylated (green) peptide. The locations of event cluster [**1**] and cluster [**2**] are highlighted in red and yellow, respectively. Measurements in 3 M LiCl and 50 mM citric acid buffered to pH 3.8 under an applied voltage of −50 mV. Data were recorded at 50 kHz sampling frequency, with a 10 kHz Bessel filter.

Encouraged by these results, we set out to apply our method to detect and quantify the extent of glycosylation in a relevant protein glycosylation reaction. We use protein-arginine rhamnosyltransferase EarP, which is known to rhamnosylate elongation factor P (EF-P), a protein that alleviates ribosome stalling in bacteria.^41^ Since a structural motif is required for rhamnosylation of small peptides,^42^ we first tested a cyclic peptide 11-mer_*Pa*, which is an l-Pro-d-Pro-cyclized fragment containing residues 28–36 of EF-P from *Pseudomonas aeruginosa* (Figure 2A). From the nanopore events, we generated a spectrum containing the *I*_ex_% and dwell time of the events, where we expect events belonging to the same peptide to cluster together. The unmodified peptide (11-mer_*Pa*_unmod) showed a major event cluster at *I*_ex_% of 89.4 ± 0.2% (Figure 2B). A second cluster at 83.9 ± 0.4 *I*_ex_% was observed constituting only 2.4% of the events, which probably reflects a contaminant in the sample, as additional peaks in the LC/MS measurements were also observed (Figure S7). The cyclic peptide includes residue Arg32, which is rhamnosylated by EarP from *P. aeruginosa* with 85% efficiency,^42^ thereby increasing the mass of the peptide by 146 Da. After enzymatic rhamnosylation using EarP, the major peptide peak shifted to higher *I*_ex_% (92.5 ± 0.8 *I*_ex_%, cluster [**1**]), reflecting the rhamnosylation of the peptide (11-mer_*Pa*_Rha). A smaller cluster of unmodified peptides (*I*_ex_% = 89.2 ± 0.5%, cluster [**2**]), was also observed (Figure 2C). In addition, we observed a cluster of events where the pore is almost fully blocked (*I*_ex_% > 95%). Although similar events can originate from intrinsic blockades of the funnel-shaped nanopore,^20^ we found they are more often detected in measurements with the rhamnosylated sample compared to the measurement with only the unmodified peptide. This indicates that these events originate from compounds used for the rhamnosylation of the peptide. The LC spectra of the rhamnosylated peptide (Figure S8) show few additional peaks, so the exact nature of this small contaminant could not be determined.

In order to quantify the extent of rhamnosylation, we used MS and nanopore measurements. The conversion can be estimated by MS using the ion intensities corrected for the relative ionization factor (RIF) of the peptides, a method that was adapted from Jewett et al.^43^ The RIF reflects the ionization ratio of the unmodified and modified peptide, which depends on the physicochemical properties of the peptides. In order to calculate the RIF, the peak area corresponding to the modified (11-mer_*Pa*_Rha) and unmodified (11-mer_*Pa*_unmod) peptides was measured for the EarP-treated sample, and the apparent percentage of modified peptides was calculated [*I*(Rha)]. Then a 1:1 mixture of the rhamnosylated and unmodified sample (Figure S9) was measured, and the apparent percentage of modified peptides was calculated [*I*(mix)]. The RIF can then be calculated using eq 1:^43^
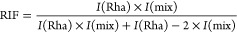
2and was determined to be 1.03 for 11-mer_*Pa* in a previous study.^42^ Based on the LC-MS results (Figures S8 and S9), the conversion was determined to be 89.8% (Table 1).

**Table 1:** Determination of the Conversion Using MS and Nanopore Measurements^a^

	%(Rha)	%(mix)	RIF	RDF	conversion
mass spec	90.1%	–	1.03^42^	–	89.8%
nanopore	87.9 ± 1.6%	41.8 ± 0.4%	–	0.89 ± 0.02	88.9 ± 1.6%

a %(Rha) is the percentage of 11-mer_*Pa*_Rha detected in the rhamnosylated sample. %(Mix) is the percentage of 11-mer_*Pa*_Rha detected in a 1:1 (m:m) mixture of 11-mer_*Pa*_Rha and 11-mer_*Pa*_unmod. RIF is the relative ionization factor of 11-mer_*Pa*_Rha, and RDF is the relative detection factor for the nanopore measurements of 11-mer_*Pa*_Rha. The conversion is the percentage of rhamnosylated peptide based on the measurement of the rhamnosylated sample and corrected by the RIF (mass spec) or RDF (nanopore).

To quantify the nanopore data, we adopted a similar strategy, where the intensity of the cluster of rhamnosylated peptide was adjusted by a relative detection factor (RDF) to correct for differences in capture frequency and detection efficiency in the nanopore. We define the RDF as
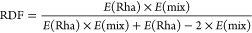
3where [*E*(Rha)] is the percentage of events in cluster [**1**] relative to cluster [**2**] in the rhamnosylated sample and [*E*(mix)] is the percentage events in cluster [**1**] relative to cluster [**2**] in the 1:1 mixture. The rhamnosylated sample and the 1:1 mixture (Figure 2D) were measured in triplicates, and an RDF of 0.89 ± 0.02 was found (Table 1), showing that the rhamnosylated peptide is detected slightly less efficiently. Both the unmodified and rhamnosylated peptide have a long dwell time in the pore at −50 mV (34.5 ± 2.0 ms and 47.4 ± 2.8 ms, respectively), indicating the difference in detection efficiency is not likely to be related to a smaller number of events detected. The dwell time decreases at higher applied voltages (Figure S10), indicating that these peptides are able to translocate across the nanopore. However, it might be a result of small modulations in the diffusivity of the glycosylated peptide. Nonetheless, we cannot exclude it might arise from the uncertainty of measuring the weight of the peptides. The extent of rhamnosylation estimated from the nanopore data is 88.9 ± 1.6% and is in good agreement with the estimation based on MS data, showing that the nanopore approach is able to accurately quantify the extent of rhamnosylation.

## Detection of Rhamnosylation in Lys-C Digested EF-P

**Figure 3:**
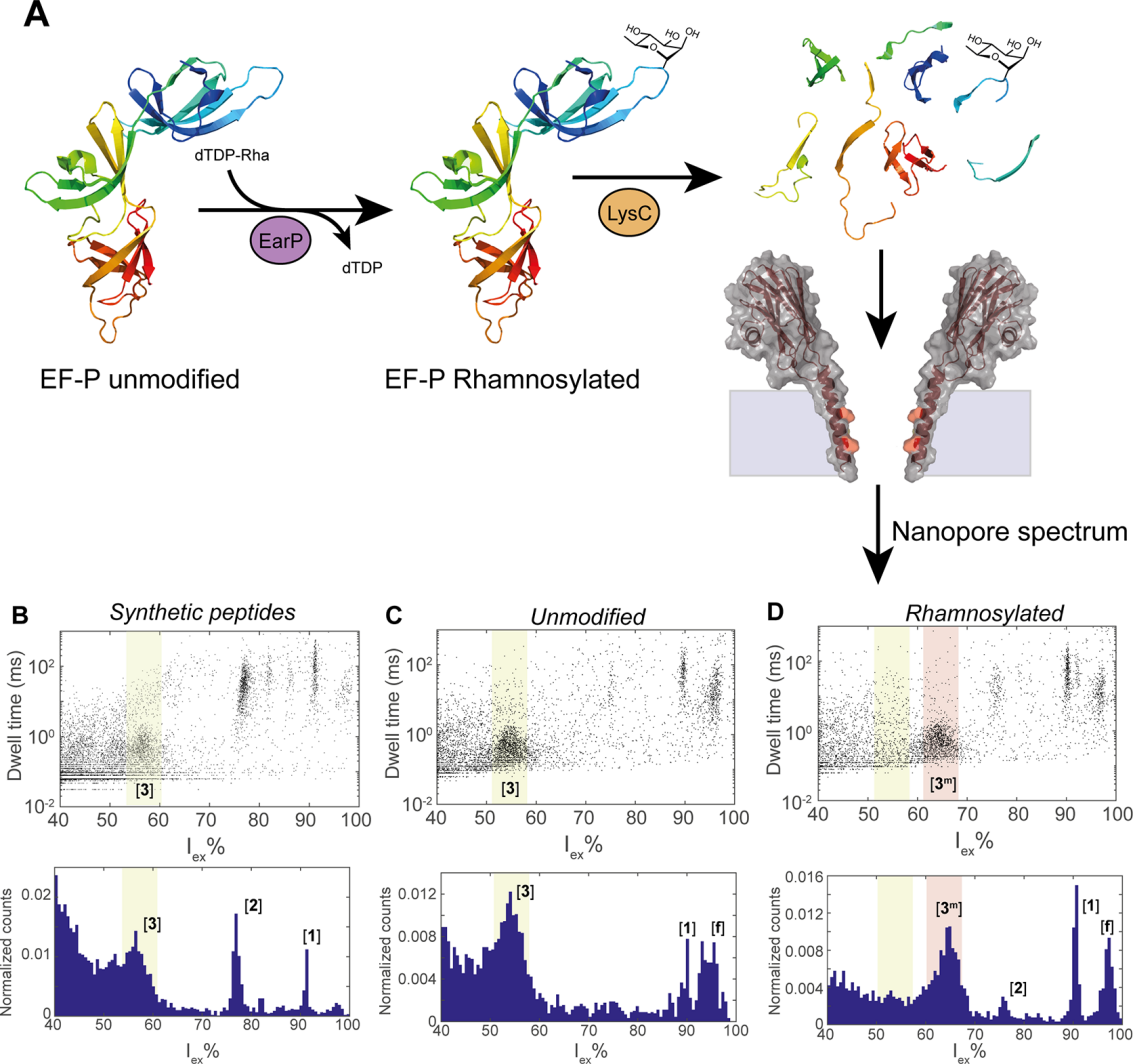
Detection of rhamnosylation in EF-P. (A) Schematic representation of the rhamnosylation and subsequent LysC digestion of EF-P. (B) Event characteristics after the addition of a mixture of synthetic peptides ([**1**], [**2**], [**3**], and [**4**]). (C) Event characteristics after addition of Lys-C digested unmodified EF-P. (C) Event characteristics after addition of Lys-C digested rhamnosylated EF-P. The location of the event clusters [**f**], [**1**], [**2**], [**3**], and [**3**^**m**^] are indicated in the *I*_ex_% histogram, and the event clusters belonging to peptide [**3**] and [**3**^**m**^] are highlighted in yellow and red, respectively. Measurements in 3 M LiCl, buffered to pH 3.8, at −50 mV applied voltage. Data were recorded with a 50 kHz sampling frequency and a 10 kHz Bessel filter.

We proceeded to quantify rhamnosylation of EF-P, the native protein that is rhamnosylated by EarP at position Arg32. The enzymatically rhamnosylated protein was first digested into a mixture of peptides using Lys-C, a protease that cleaves specifically after lysine residues (Figure 3A). The full digestion of EF-P yields four peptides (named [**1**], [**2**], [**3**], and [**4**]) in the mass range of 500–2000 Da (Table 2), including peptide [**3**] (SGRNAAVVK), containing residue Arg32. We measured the nanopore signals from the synthetic peptides (peptides [**1**], [**2**], [**3**], and [**4**], Figure 3B, Figure S12) to confirm that our nanopore system would be able to detect these peptides. Peptides [**1**], [**2**], and [**3**] were detected as individual well-defined event clusters, while peptide [**4**] was not detected in our nanopore system, most likely due to the small size of the peptide. Then, we measured the nanopore spectra of unmodified EF-P and rhamnosylated EF-P, which showed event clusters at similar locations as the synthetic peptides. One additional cluster [**f**], corresponding to <5% of the total events, was also observed (Figure 3C,D), most likely reflecting larger peptides (>2000 Da) in the sample, resulting from the digestion of EF-P by Lys-C (Tables S2 and S3). We could not detect peptide [**4**] in the samples. Nonetheless, this peptide was also not detected in the MS measurements of the samples (Tables S2 and S3), suggesting it might not be produced by the enzymatic reaction or might escape detection due to its small size. Notably, the peak corresponding to the unmodified EF-P (cluster [**3**], *I*_ex_% = 54.8 ± 1.3) shifts to a higher excluded current (64.6 ± 0.2 *I*_ex_%) after rhamnosylation (cluster [**3**^**m**^]), in line with the expected mass increase of the peptide. In addition, we prepared a mixture of unmodified and rhamnosylated EF-P in a 1:1 volume ratio and performed Lys-C digestion. The mixture was then measured by the nanopore, resulting in a spectrum containing both cluster [**3**] (*I*_ex_% = 57.6%) and cluster [**3**^**m**^] (*I*_ex_% = 64.4%, Figure S12), and the presence of peptide [**3**] and [**3**^**m**^] in the sample was confirmed by MS measurements (Tables S2 and S3). Finally, we estimated the yield of rhamnosylation using the nanopore measurement. We calculated the percentage of events in cluster [**3**^**m**^] relative to the total events in cluster [**3**] and [**3**^**m**^] (Figure 3D), from which the yield of rhamnosylation of 90.2 ± 3.4% could be estimated. Considering the RDF of 0.89 (Table 1), we found a 91.1 ± 3.1% conversion, which is not significantly different from the 92.6% conversion of EF-P estimated by measuring intact proteins using MS (Figure S13). Thus, we conclude that our nanopore system is able to detect and quantify rhamnosylation reactions on EF-P in a complex mixture of peptides.

**Table 2:** Expected Peptides in the Mass Range 500–2000 after Lys-C Digestion of EF-P and Rhamnosylated EF-P

peptide	mass (Da)	position in EF-P	sequence
[**1**]	1350.7	43–55	NLLTGAGTETVFK
[**2**]	1096.7	60–68	LEPIILDRK
[**3**^**m**^]	1047.5	30–38	SGR{Rha}NAAVVK
[**3**]	901.5	30–38	SGRNAAVVK
[**4**]	608.3	25–29	AEFNK

In this work, we have shown that biological nanopores can be adapted toward the detection and quantification of monosaccharide modifications on peptides and native proteins. Earlier work on the detection of glycosylation in biological nanopores mainly focused on model peptides^31^ as well as branched glycan chains on specific folded proteins.^16^ Although the detection of peptides from proteolytic fragment has been recently reported,^38,44^ the detection of hydrophilic or glycosylated generic peptides has not been reported. This is most likely the result of their rapid translocation through the pore, which complicates their detection. In this work, we show that a nanopore with an aromatic constriction together with high electrolyte concentrations and a low pH increases the dwell time for the translocation of hydrophilic peptides and glycopeptides, allowing their selective detection. In addition, we accurately detected and quantified the extent of rhamnosylation on cyclic peptides as well as on the proteolytic peptides from native EF-P protein. Therefore, our nanopore approach can identify and quantify glycosylation on generic proteins, regardless of their size and structure. Although at the moment the nanopore approach cannot compete with the resolution of MS, nanopores have unique advantages as they can be integrated into low-cost, portable devices that can are compatible with single-molecule detection.^45,46^ Finally, it is important to note that the nanopore signal not only depends on the volume of the peptide but also contains information about the chemical interaction with the analyte, which might be further used to identify peptides that are difficult to study using MS.

## Supporting Information Available

Materials and methods, Figures S1–S9, Tables S1 and S2, including HPLC and MS measurements of the glycopeptides, cyclic peptides, and EF-P and a description of the solid-phase synthesis of the 9mer peptide (PDF)

## Notes

Data availability: The authors declare that the data supporting the findings of this study are available within the article and its Supporting Information or from the corresponding authors upon reasonable request.
