# Supplementary material for: Quantification of Protein Glycosylation Using Nanopores
Source: Nano Lett. 2022 Jun 29;22(13):5357–64. doi: 10.1021/acs.nanolett.2c01338 (PMC9284675; doi:10.1021/acs.nanolett.2c01338)
Supplement: Supplementary file 1 — nl2c01338_si_001.pdf [file nl2c01338_si_001.pdf]

Quantification of Protein Glycosylation using Nanopores

Roderick Corstiaan Abraham Versloot[1], Florian Leonardus Rudolfus Lucas[1], Liubov Yakovlieva[2], Matthijs Jonathan Tadema[1], Yurui Zhang[3], Thomas M. Wood[3], Nathaniel I. Martin[3], Siewert J. Marrink[1], Marthe T. C. Walvoort*[2], Giovanni Maglia*[1]

[1] Groningen Biomolecular Sciences and Biotechnology Institute, University of Groningen, 9747AG Groningen, The Netherlands

[2] Chemical Biology division, Stratingh Institute for Chemistry, University of Groningen, 9747AG Groningen, The Netherlands

[3] Biological Chemistry Group, Institute of Biology Leiden, Leiden University, 2333 BE Leiden, The Netherlands

* Correspondence: g.maglia@rug.nl, m.t.c.walvoort@rug.nl

Table of contents

Materials and Methods	Page 3-8
Figure S1-S3: LC-MS analysis of glycosylated 9mer peptides and mixture thereof	Page 9-11
Figure S4: Initial screening of other FraC mutants for glycopeptide detection	Page 12
Figure S5: Example ionic current traces of equimolar glycopeptide measurements	Page 13
Figure S6: Additional current blockades of the glycopeptides in FraCG13F	Page 14
Figure S7: LC-MS measurement of 11mer_*Pa*_unmod	Page 15
Figure S8: LC-MS measurement of 11mer_*Pa*_rham	Page 16
Figure S9: LC-MS measurement of a 1:1 (v:v) mixture of 11mer_*Pa*_unmod and 11mer_*Pa*_rham	Page 17
Figure S10: Peptide measurements at higher applied voltage	Page 18
Figure S11: SDS-PAGE of Rha-EFP purification fractions	Page 19
Figure S12: Nanopore spectrum of synthetic EF-P peptides.	Page 20
Figure S13: Mass spec traces of EF-P and Rhamnosylated EF-P	Page 21
Table S1: Event frequency analysis of glycopeptides in 1M KCl	Page 22
Table S2: Detected EF-P peptides in the MS measurement of LysC-digested EF-P	Page 23
Table S3: Detected EF-P peptides in the MS measurement of LysC-digested rhamnosylated EF-P	Page 23
Solid-phase peptide synthesis of the 9mer peptide	Page 24
Figure S13: Analytical HPLC traces of 9mer peptides	Page 25

Materials and Methods

Chemicals

Potassium chloride (>99.5%), sodium chloride (>99.5%), lithium chloride (>99%), imidazole (>99%), dodecyl-β-D-maltoside (DDM, >99%), TRIS hydrochloride PUFFERAN® (≥99%), urea, LB medium and 2YT medium were purchased from Carl Roth. Bistrispropane (BTP, >99%), N,N-dimethyldodecylamine N-oxide (LDAO, >99%), pentane (>99%) and n-hexadecane (>99%) were purchased from Simga-Aldrich. Citric acid (anhydrous, 99.6%) was obtained from Acros Organics. 1,2-Diphytanol-sn-glycero-3-phophocholine (DPhPC) lipids and sphingomyelin were obtained from Avanti. Thymidine-5'-diphosphate-L-rhamnose (TDP-Rha) was purchased from Carbosynth. Ampicillin and isopropyl-β-D-1-thiogalactopyranoside (IPTG) were purchased form Fisher Scientific. The 9mer peptide was prepared using solid-phase peptide synthesis (see Supplementary Information), and the cyclic peptide 11-mer_*Pa* was prepared as previously described.[1]

FraC monomer expression and purification

Plasmid containing the FraC gene was transformed into BL21(DE3) cells using electroporation. The transformed cells were grown overnight at 37 ºC on LB agar plates supplemented with 1% glucose and 100 µg/ml ampicillin. On the next day, the colonies were pooled together and resuspended and grown in 200 mL 2YT medium at 37 ºC until the OD_{600} reached a value of 0.6-0.8. At this point, the expression was induced by the addition of 0.5 mM IPTG and the culture was incubated overnight at 25 ºC. Afterwards, the cells were pelleted by centrifugation at 4000 rpm for 15 minutes and the cell pellets were stored at -80 ºC for at least 30 minutes. For protein purification, cell pellets of 100 ml culture were resuspended in 20 ml lysis buffer, containing 150 mM NaCl, 4M Urea, 20mM imidazole and 15 mM Tris buffered to pH 7.5, supplemented with 1 mM $MgCl_2$, 0.2 units/ml DNase1 and approximately 1 mg of lysozyme. The mixture is incubated for 30 minutes at RT and subsequently sonicated using a Branson Sonifier 450 (2 minutes, duty cycle 30%, output control 3) to ensure full disruption of the cells. Cell debris is pelleted by centrifugation at 6000 rpm for 20 minutes and the supernatant is

carefully transferred to a fresh falcon tube. Meanwhile, 200 µl of Ni-NTA bead solution is washed with wash buffer, containing 150 mM NaCl, 20 mM imidazole and 15 mM Tris buffered to pH 7.5. The beads are added to the supernatant and incubated at room temperature for 5 minutes. Afterwards, the solution is loaded on a Micro Bio-Spin column (Bio-Rad) and washed with 5 ml of wash buffer. The bound protein is eluted in fractions of 200 µl of elution buffer (150 mM NaCl, 300 mM imidazole, 15mM Tris buffered at pH 7.5). The presence of FraC monomers was detected using SDS-PAGE.

FraC oligomerisation

FraC monomers were incubated with DPhPC:Sphingomyelin (1:1) liposomes in a 1:10 protein:lipid mass ratio at 37ºC for 30 minutes. Afterwards, the liposomes were disrupted by the addition of 0.6% LDAO and incubation at room temperature for 5 minutes. The sample was diluted 20 times in wash buffer containing 150 mM NaCl, 20 mM Imidazaole and 15 mM Tris buffered to pH 7.5 supplemented with 0.02% DDM. Meanwhile, 200 µl of Ni-NTA bead solution is washed with wash buffer and subsequently added to the dissolved liposomes. The mixture is incubated at room temperature for 5 minutes and loaded on a Micro Bio-Spin column (Bio-Rad). The column is washed with 5 mL of wash buffer and oligomers are eluted in 200 µl elution buffer containing 1M imidazole, 150 mM NaCl and 15 mM Tris buffered to pH 7.5 supplemented with 0.02% DDM. Oligomers can be stored at 4ºC for several weeks.

Single-channel recordings

Two fluidic compartments are separated by a polytetrafluoroethylene (Teflon) film (Goodfellow Cambridge Ltd) with a thickness of 25 µm, containing an aperture of approximately 100 µm in diameter. First, 10 µl of a 5% hexadecane solution in pentane is applied to the aperture and the pentane is left to evaporate shortly. Afterwards, both compartments are filled with 400 µl buffer and 10 µl of a 10 mg/ml DPHPC solution in pentane is added on top of the buffer solution. The chamber is left to evaporate the pentane and an Ag/AgCl electrode is placed in each compartment as to make contact with the buffer solution. Planar lipid bilayers were formed by repeatedly lowering and raising the buffer solution until a stable lipid bilayer was formed. FraC

nanopores were added to the *cis*-compartment and the lipid bilayer was reformed until a single channel was present. Presence of a single channel and its orientation were confirmed by the IV characteristics of the pore. A two-minute blank was recorded and afterwards substrate was added to the *cis*-compartment.

Data acquisition

The ionic current was recorded using a Digidata 1440A (Molecular Devices) connected to an Axopatch 200B amplifier (Molecular Devices). All data is recorded with a sampling frequency of 50 kHz and with a Bessel filter of 10 kHz. The data is then digitally filtered using a 5 kHz Gaussian low-pass filter prior to the event detection.

Event detection

First, using Clampfit software, the full-point histogram of the ionic current trace was taken in order to determine the open pore current (I_o) and the open pore noise (σ_{I_0}). A Gaussian around the open pore current was fitted to determine the peak centre (I_o) and standard deviation (σ_{I_0}). Then, events were detected using a threshold search with a threshold of 5*σ_{I_0} and with a minimum duration of 50 µs. The excluded current percent (I_{ex}%) was calculated using $I_{ex}\% = \left(\frac{\Delta I_B}{I_0}\right) * 100\%$, where ΔI_B (=$I_O - I_B$) is the magnitude of the current blockade.

Glycopeptide dwell time analysis

After event detection, the average dwell time of the peptides was estimated for each salt concentration in triplicates. First, the I_{ex}% range was determined for each peptide cluster: 71 to 78% for 9mer_2Glc, 62 to 68% for 9mer_1Glc and 56 to 61% for 9mer_unmod. A log-normal distribution was fitted through the dwell time histogram to obtain the mean dwell time of each peptide cluster. The standard deviation is calculated between the three individual measurements in different nanopores.

Glycosylation and purification of the 9mer peptides (ANVTLNTAG and ANVTLNTTG)

Reaction mixtures (total 5.4 mL) consisted of 0.5 mM 9mer peptide (~2.5 mg) (from 10 mM DMSO stock to keep DMSO content low) and 2.5 mM UDP-Glc in the reaction buffer (50 mM HEPES, 100 mM NaCl, 10% glycerol, pH 7.5). Reactions were initiated by the addition of 10 µM ApNGT[2] and incubated at room temperature overnight. The glycosylation of ANVTLNTTG was pushed over the next five days by addition of total extra 7.3 mg of UDP-Glc and 10 µM of ApNGT until ~90% conversion to the di-glycosylated product was reached. To prepare the samples for LCMS analysis, an aliquot of the reaction mixture was diluted 10-fold and analyzed with RP-LCMS (1 µL injection, Acquity UPLC HSS T3 column (Waters, 2.1×150 mm, 1.8 µm) in combination with eluents A (0.1% formic acid in H_2O) and B (0.1% formic acid in acetonitrile), 20 min run (flow rate 0.3 mL/min) with a linear gradient from 5% to 95% of B in 13 min with subsequent increase to 95% B for 3min and return to 5% B). Before anion exchange purification, reaction mixtures were filtered through a 0.2 µm filter. The resulting peptides were separated from the ApNGT, UDP and UDP-Glc by strong anion exchange on FPLC ÄKTA system (GE Healthcare). For this, 1 mL of the reaction mixture (~ 0.2 mM peptide concentration) was applied on a Q FF column (5 mL, GE Healthcare) with a flow rate of 1.5 mL/min. The column was eluted with the linear gradient from 0 to 60% Buffer B (0.9 M NH_4HCO_3) in ten column volumes, with subsequent increase to 100% in four CV. Elution was monitored with UV (214 nm – peptide and 280 nm – UDP, UDP-Glc). The fractions containing glycosylated peptides (first 5-6 min of the elution) were pooled and freeze-dried. Dried samples were reconstituted in DMSO to prepare stocks for the nanopore analysis.

Rhamnosylation and purification of the 11mer_*Pa* peptide

To prepare rhamnosylated 11mer-L-Pro-D-Pro_*Pa*, 2.7 mM 11mer_*Pa* (1 mg), 6.1 mM TDP-Rha (1.2 mg) and 41 µM EarP were incubated at room temperature for two days in the reaction buffer (20 mM Tris, 100 mM NaCl, pH 8). The reaction was pushed to full conversion over the next three days by addition of total extra 1 mg of TDP-Rha and 24 µM of EarP until ~90%

conversion was reached. Subsequently, the reaction mixture was diluted to a 0.5 mM concentration of Rha-11mer, applied to an Amicon spin filter (MWCO 10 kDa, 15 mL, Millipore) and centrifuged at 5000 x *g* to remove the enzyme. The resulting solution was further purified from TDP and TDP-Rha by strong anion exchange on FPLC (ÄKTA system, GE Healthcare). For this, 0.25 mL of the reaction mixture was applied on Q FF column (5 mL, GE Healthcare) with flow rate 1 mL/min in five column volumes (CV). The column was eluted with the linear gradient from 0 to 10% Buffer B (1M NH_4HCO_3) in two CV with subsequent increase to 100% in four CV. Elution was monitored with UV (214 nm – peptide and 280 nm – TDP, TDP-Rha). The fractions containing rhamnosylated 11mer_*Pa* peptide were pooled and freeze-dried. Residual buffer salts were removed by desalting with PD-10 desalting columns (GE Healthcare). Desalted fractions of Rha-11mer were freeze-dried and aqueous stock of 0.5 mM was prepared for the nanopore studies.

Quantification of rhamnosylation on peptides using the nanopore

After event detection, the I_{ex}% spectra of the three measurements were first realigned to correct for small shifts in the baseline. After baseline correction, a Gaussian mixture model was used to detect the event clusters. We found that the clusters are best detected in the I_{ex}% vs event noise (I_{SD}) spectrum, where I_{SD} is the fluctuation of the ionic current during the event. From the fitting of the Gaussian mixture model, the location and distribution of the event clusters in the I_{ex}% vs I_{SD} spectrum was obtained. For each detected event cluster, the center (μ_1) and the spread (σ_1) in I_{ex}% and the center (μ_2) and spread (σ_2) in I_{SD} was determined. Then, for each event cluster the events that satisfy both (μ_1-σ_1) > I_{ex}% > (μ_1+σ_1) and (μ_2-σ_2) > I_{SD} > (μ_2+σ_2) were counted to obtain the number of events belonging to the rhamnosylated peptide (n_{Rha}) and the unmodified peptide (n_{unmod}). In addition, the events that satisfy the same equation in the blank measurement were subtracted to reduce the effect of intrinsic current blockages. The percentage of rhamnosylation in the sample is estimated as: $n_{Rha}/(n_{Rha}+n_{unmod})*100\%$. These values are then used in equation 2 to calculate the RDF. Finally, the conversion is calculated using:

$$Conversion = \frac{\frac{E(Rha)}{RDF}}{\frac{E(Rha)}{RDF}+(100-E(Rha))} \qquad (4)$$

The standard deviation is calculated between the three individual measurements in different nanopores.

Rhamnosylation and purification of EF-P

To prepare rhamnosylated EF-P, 12 μM of EF-P (after His_6-SUMO-tag cleavage, as described in[3]) was incubated with 2 μM of EarP-His_6-SUMO and 100 μM TDP-Rha in the reaction buffer (20 mM Tris, 500 mM NaCl, pH 8) overnight at room temperature. The next day, Ni-affinity chromatography was used to isolate Rha-EFP. Briefly, the reaction mixture was incubated with Ni-NTA resins for 1.5 h at 4 ^oC with gentle shaking. The resulting suspension was allowed to pass through the gravity column and the flow-through was collected. Next, the resin was washed once with lysis buffer (20 mM Tris, 500 mM NaCl, pH 8), followed by washing buffer 1 in two steps (20 mM Tris, 500 mM NaCl, 15 mM imidazole, pH 8), and washing buffer 2 in two steps (20 mM Tris, 500 mM NaCl, 30 mM imidazole, pH 8). This was followed by elution with elution buffer (20 mM Tris, 500 mM NaCl, 400 mM imidazole, pH 8). Analysis of the purification fractions with SDS-PAGE (**Figure S7**) indicated that Rha-EFP was present in the flow-through, lysis buffer wash, and washing buffer (1 and 2) fractions. To prepare the Rha-EF-P sample for the nanopore analysis, fractions containing the protein were pooled, diafiltrated, and concentrated with Amicon spin filter to 6.5 mg/mL (0.3 mL total). The purity of the sample was confirmed with intact protein MS analysis (**Figure S8**).

Lys-C digestion of EF-P and rhamnosylated EF-P

200 ng of protein was dissolved in 180 µl buffer containing 100 mM Tris, buffered to pH 8.0. Then we add 4 µg of Lys-C, yielding a 1:50 enzyme : protein mass ratio and the sample is subsequently incubated overnight at 37 ^oC. On the next day, an Amicon filter with a molecular weight cut-off of 3000 Da was used, to eliminate the protease and any undigested protein from the sample. The sample is stored at -20 ^oC until use.

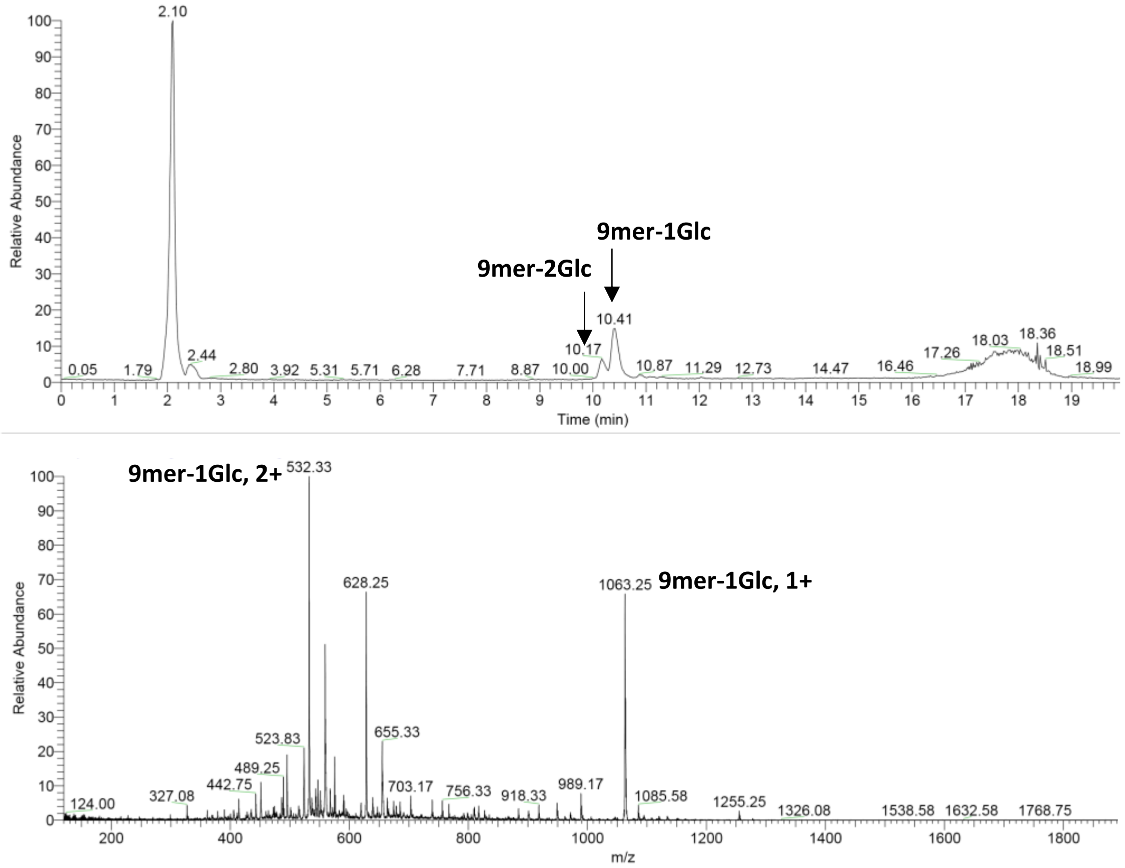

Figure S1. LCMS trace of 9mer-1Glc (ANVTLNTAG, glycosylation site underlined) after anion exchange purification. The peptide elutes after 10.41 minutes in the HPLC measurement (top) and is subsequently detected in MS (bottom). A small amount of 9mer-2Glc (10.17 min) was also detected in the sample.

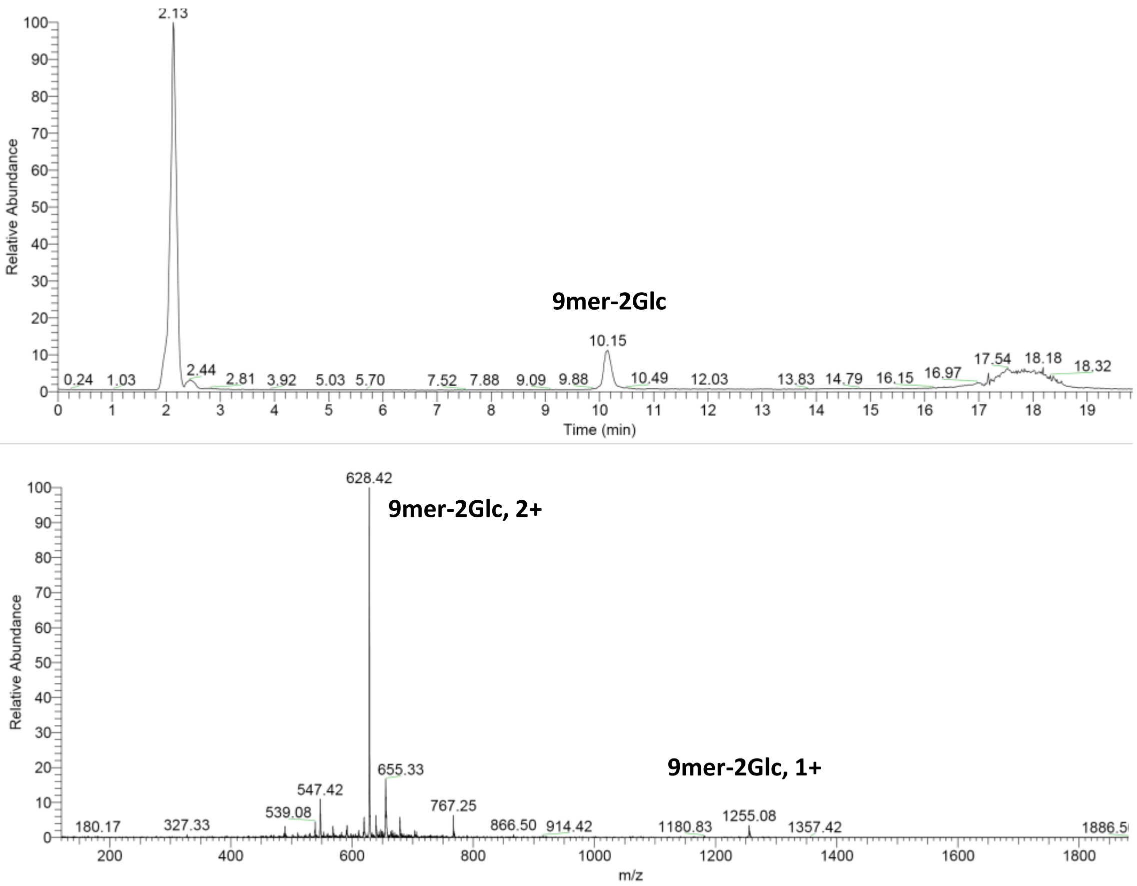

Figure S2. LCMS trace of 9mer-2Glc (A<u>N</u>VTL<u>N</u>TTG, glycosylation site underlined) after anion exchange purification. The peptide elutes after 10.15 minutes in the HPLC measurement (top) and is subsequently detected in MS (bottom).

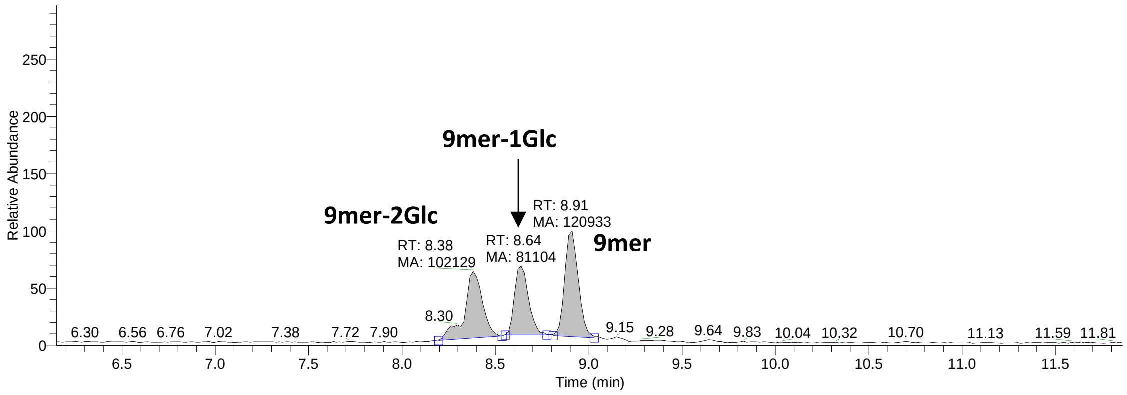

Figure S3. The LC trace of the peptide mixture containing equal volumes of the non-modified 9mer (8.91 min), mono-glycosylated 9mer (8.64 min) and di-glycosylated 9mer (8.38 min) from the stock solutions used in the nanopore analysis. RT = retention time, MA = elution peak area. *Differences in the peptide retention times compared to LCMS traces above are due to the different LCMS method that was used in this analysis.

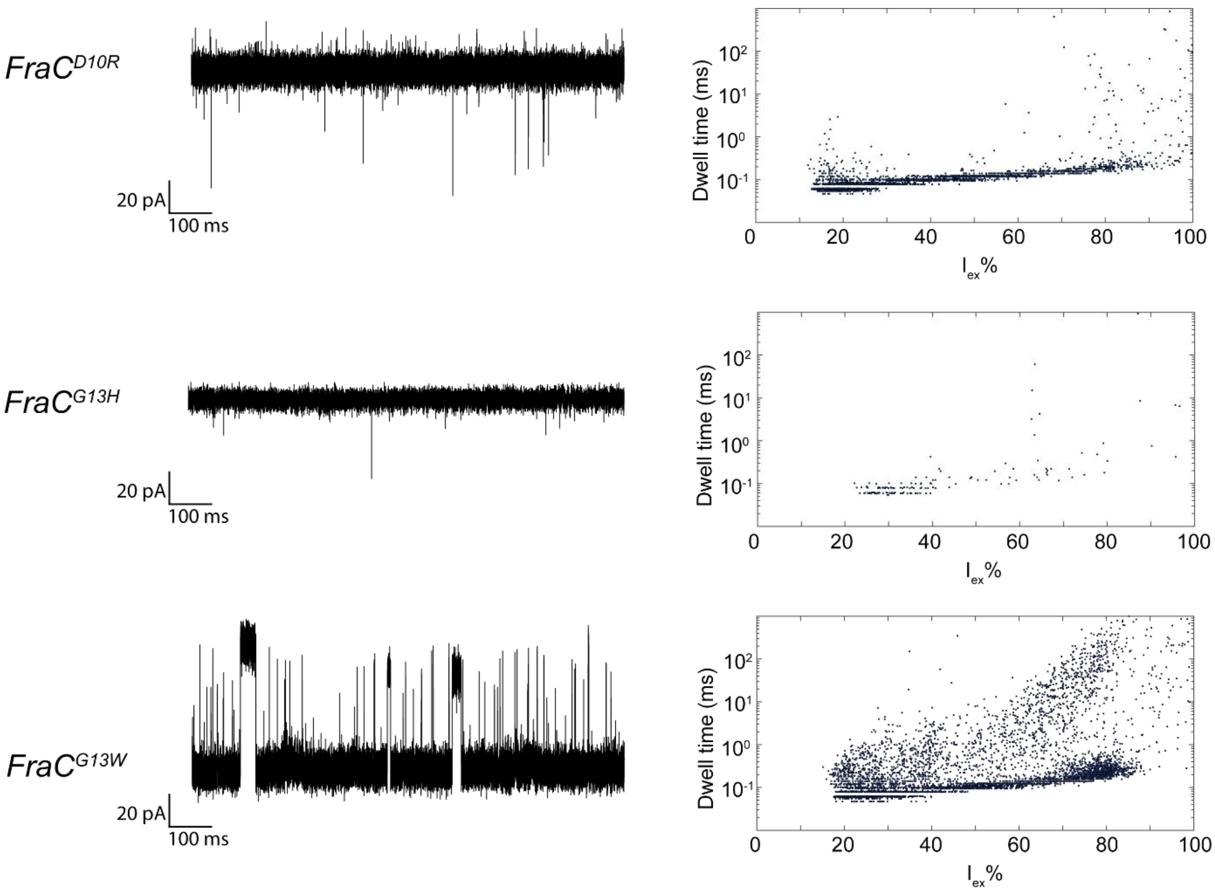

Figure S4: Initial screening of other FraC mutants for glycopeptide detection. Ionic current traces (left) and event characteristics (right) of measurements in FraC mutants with single glycopeptides. Top panel: 30 µM of 9mer_2Glc added to a FraC^{D10R} nanopore, measured at +100 mV applied voltage. Middle panel: 10 µM of 9mer_2Glc added to a FraC^{G13H} nanopore, measured at +50 mV applied voltage. Bottom panel: 7.5 µM of 9mer_2Glc added to a FraC^{G13W} nanopore, measured at -70 mV applied voltage.

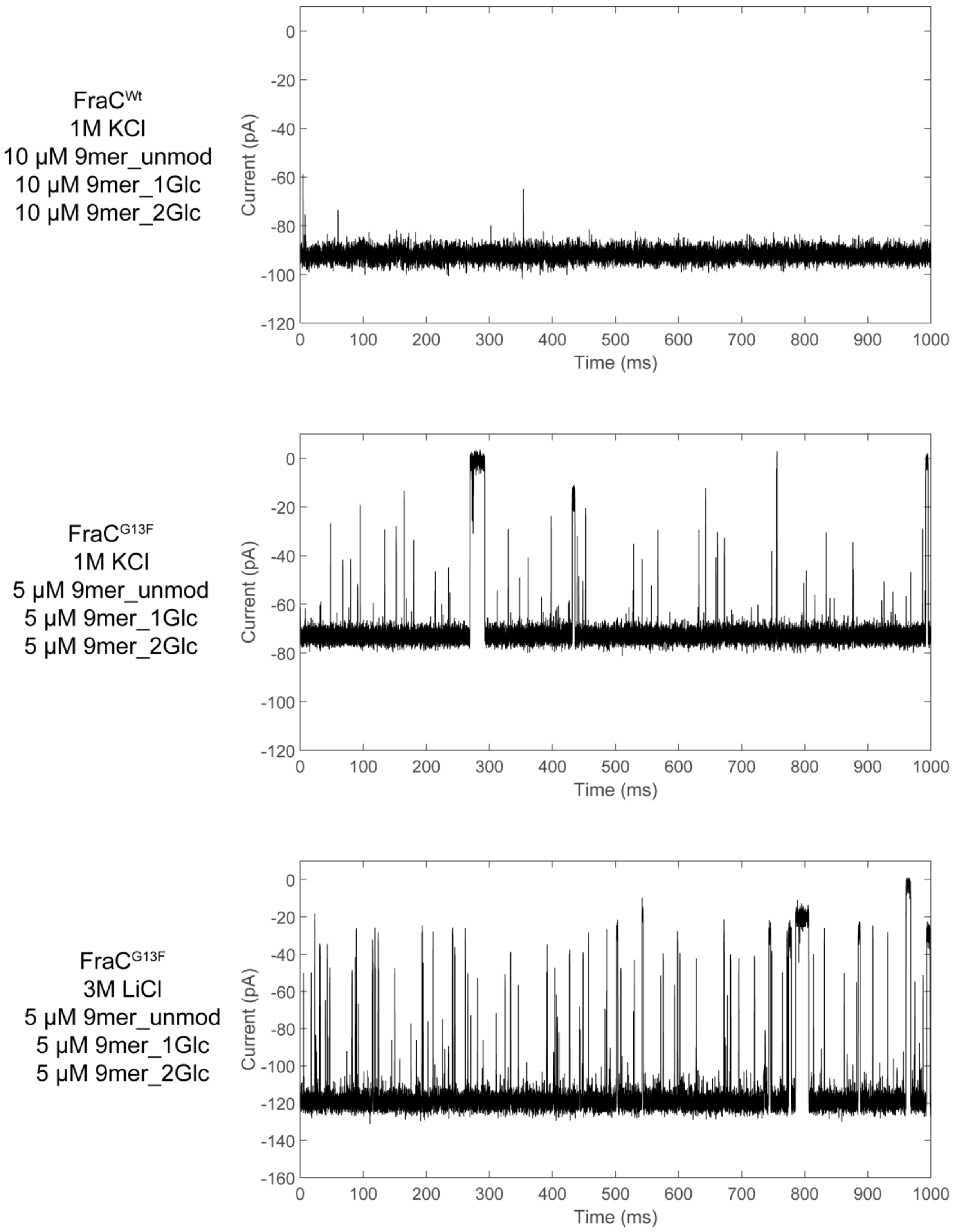

Figure S5: Example ionic current traces of equimolar glycopeptide mixture measurements. One second traces with ionic current blockades for FraC^{Wt} in 1M KCl (top), FraC^{G13F} in 1M KCl (middle) and FraC^{G13F} in 3M LiCl (bottom) after the addition of a equimolar mixture of 9mer_unmod, 9mer_1Glc and 9mer_2Glc. Measurements under an applied voltage of -50 mV, with 50 kHz sample frequency, 10 kHz Bessel. The data was afterwards filtered additionally using a 5 kHz digital filter.

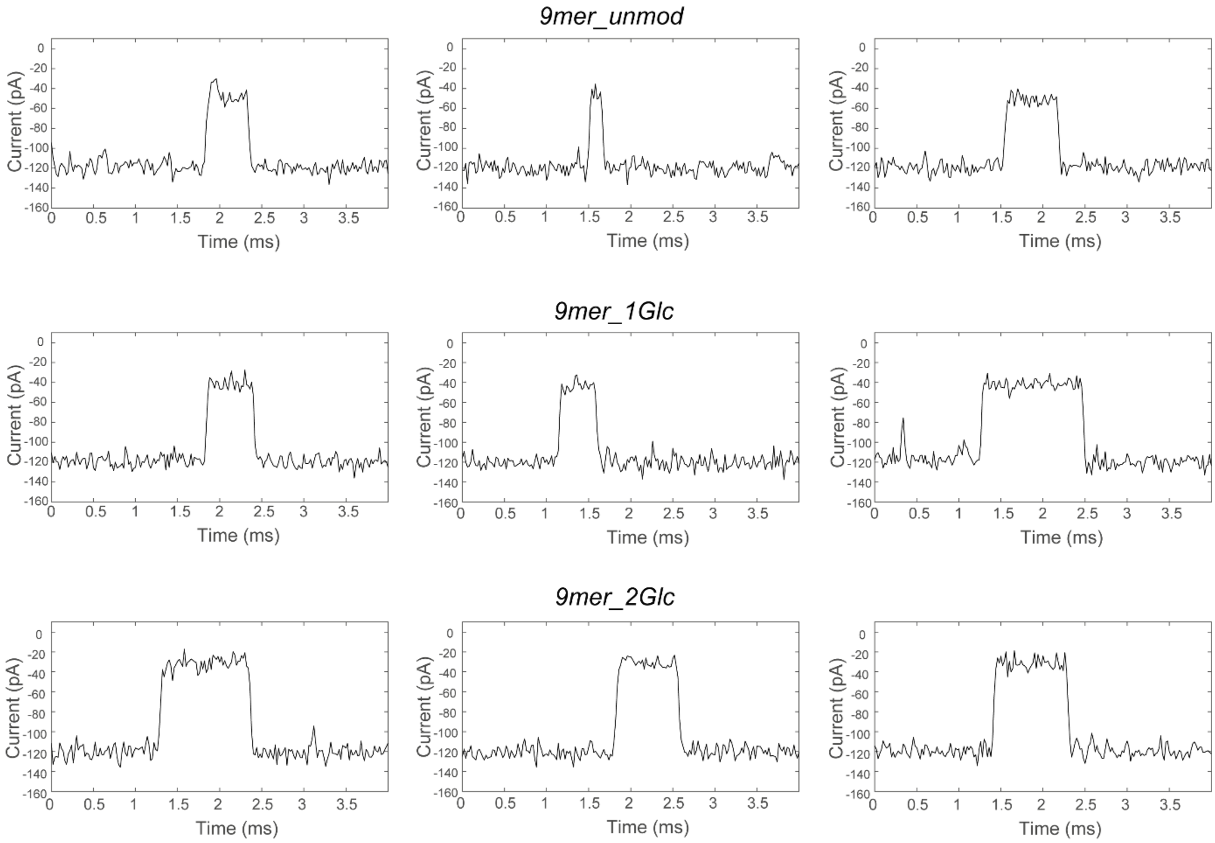

Figure S6: Additional current blockades of the glycopeptides in FraC^{G13F} in 3M LiCl solution. For each peptide three representative ionic current blockades are shown. Data acquired after the addition of a equimolar mixture of 9mer_unmod, 9mer_1Glc and 9mer_2Glc to FraC^{G13F} pores in 3M LiCl, 50 mM citric acid buffered to pH 3.8. Measurements under an applied voltage of -50 mV, with 50 kHz sample frequency, 10 kHz Bessel filter.

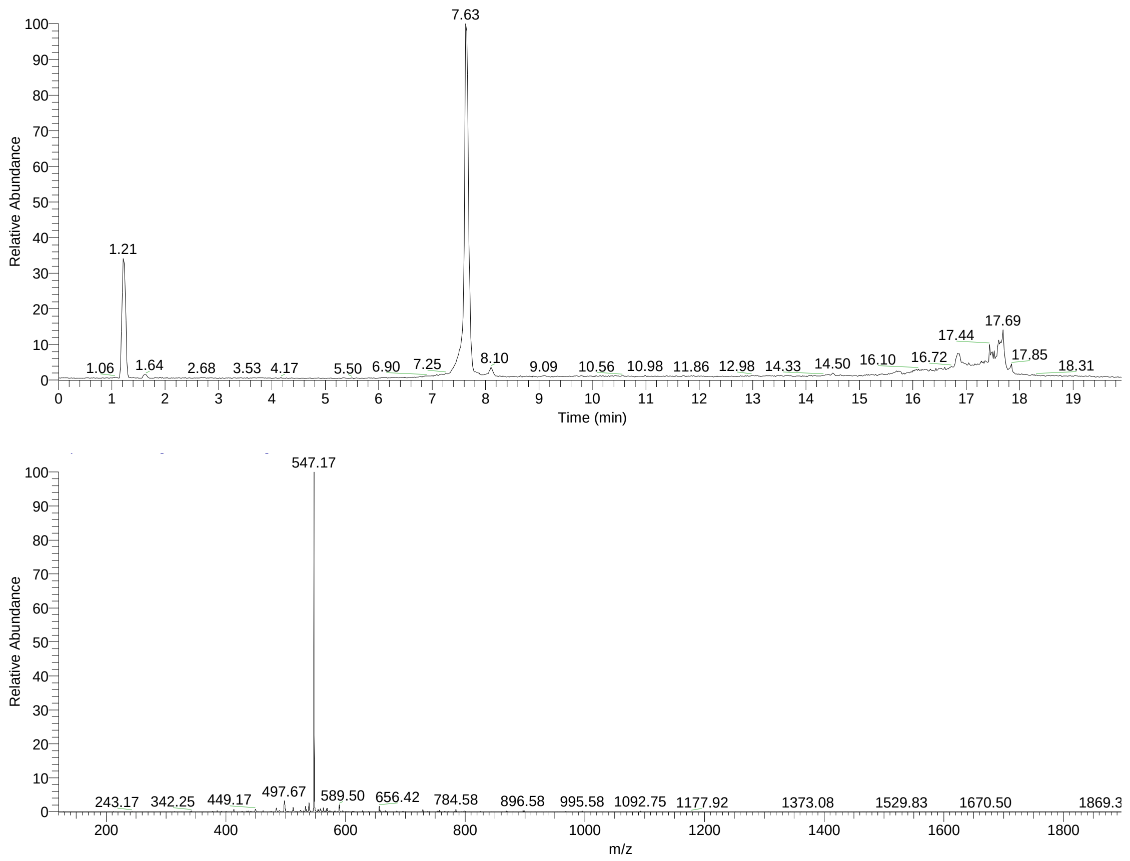

Figure S7. The LCMS trace of the non-modified 11mer_*Pa* peptide. The peptide elutes after 7. 63min in the HPLC measurement (top) and is subsequently detected in MS (bottom). A tiny peak at 8.10 minutes is visible in the HPLC measurement, that might belong to a contamination in the sample.

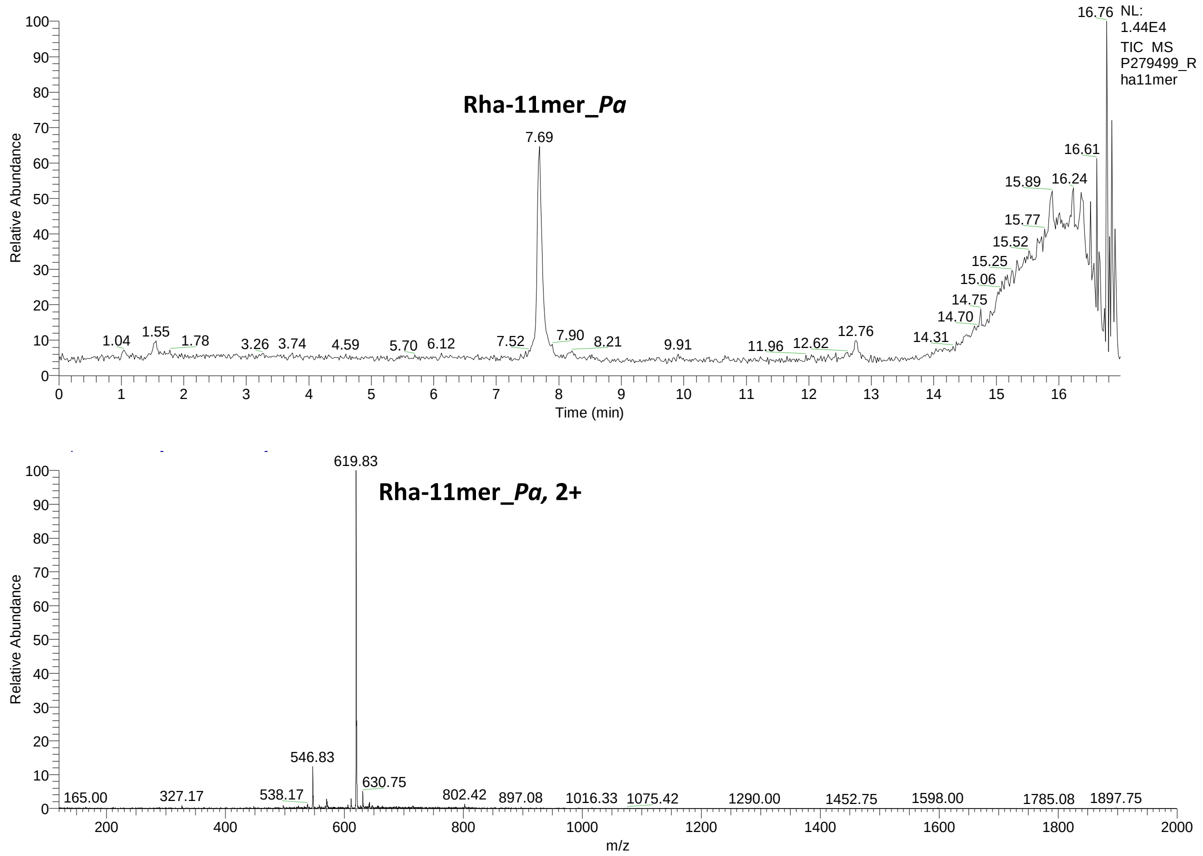

Figure S8: The LC-MS trace of Rha-11mer_*Pa* after anion exchange purification. LC chromatogram (top) showing the elution of 11mer_*Pa* at 7.69 minutes and mass spectrum of the elution peak at 7.69 minutes (bottom).

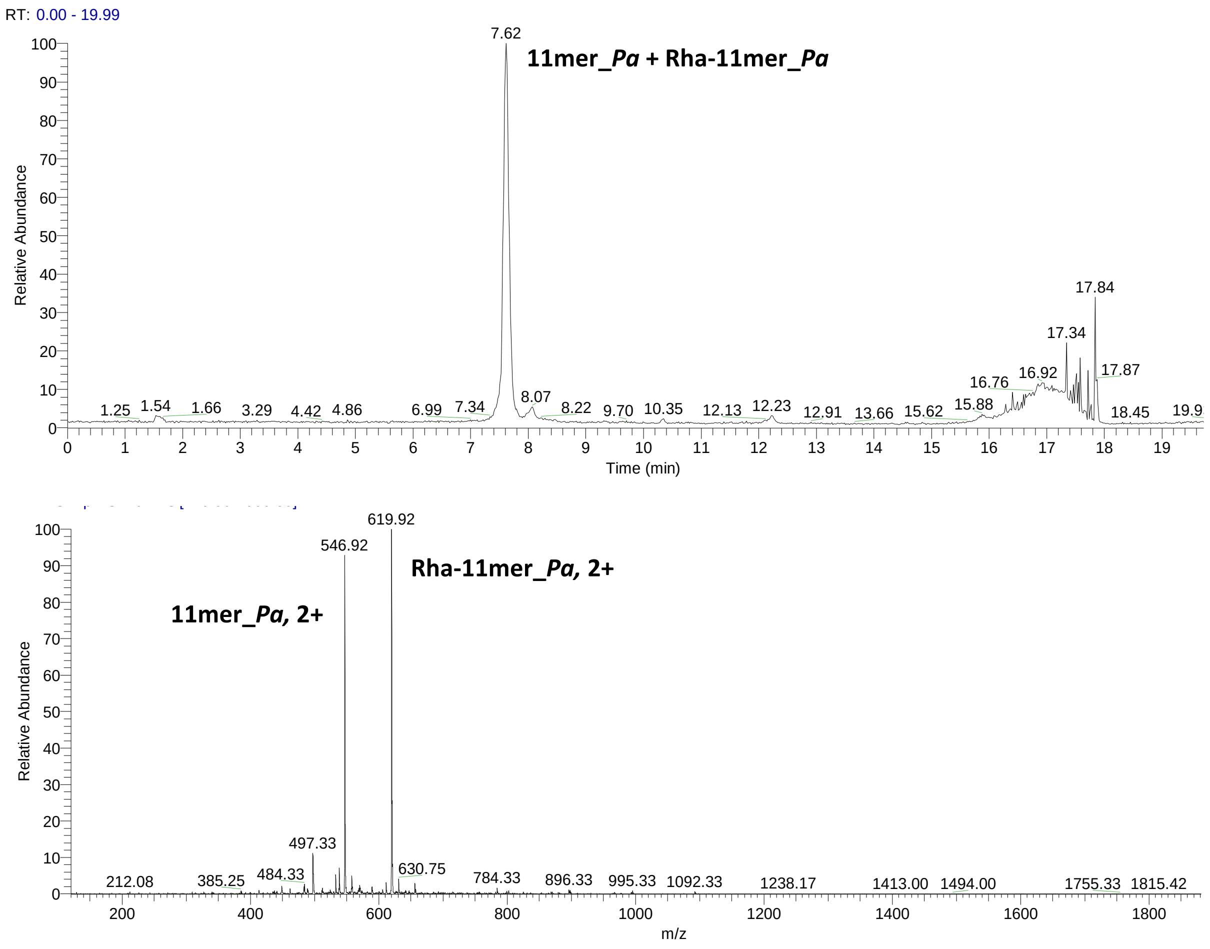

Figure S9: The LC-MS trace of a 1:1 (v:v) mixture of 11mer_*Pa* and Rha-11mer_*Pa* stock solutions used in the nanopore measurements. LC chromatogram (top) showing the co-elution of 11mer_*Pa* and Rha-11mer_*Pa* at 7.62 minutes and mass spectrum of the elution peak at 7.62 minutes (bottom).

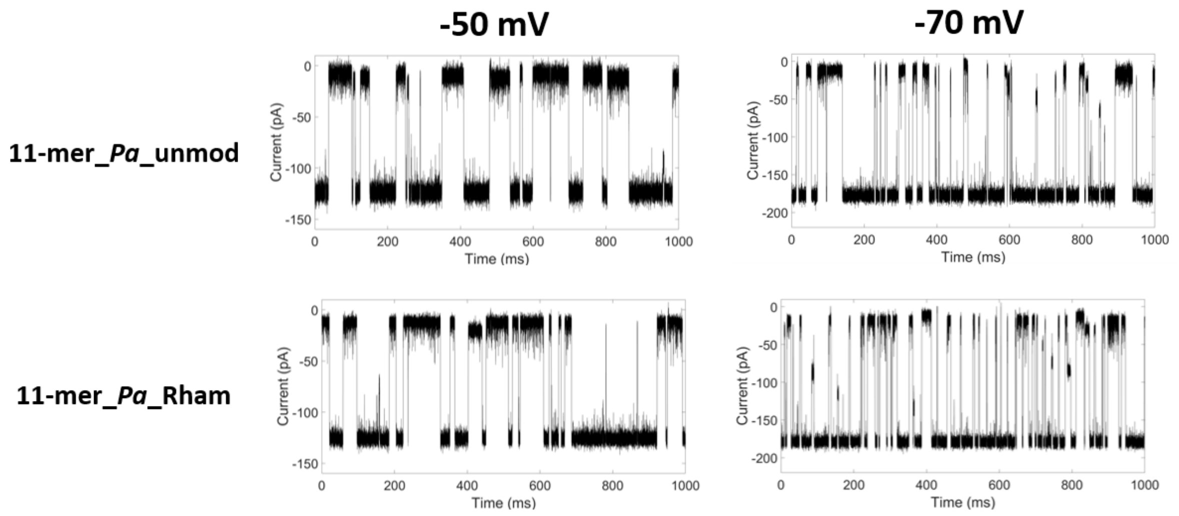

Peptide	t_{dwell} (-50 mV), ms	t_{dwell} (-70 mV), ms
11-mer_Pa_unmod	35.4 ± 2.0	8.6 ± 1.2
11-mer_Pa_rham	47.4 ± 2.8	10.5 ± 0.8

Figure S10: Faster peptide translocation at higher voltage. Representative ionic current of 11-mer_*Pa*_unmod (top) and 11-mer_*Pa*_rham (bottom) at -50 mV and -70 mV applied voltage. The table shows the average dwell time calculated by exponential fitting to the dwell time histogram. The error reflect the 95% confidence interval of the exponential fitting. The dwell time decreases significantly at higher applied voltage, indicating translocation of the peptides. Measurements in 3M LiCl, 50 mM citric acid buffered to pH 3.8, with 50 kHz sample frequency, and a 10 kHz Bessel filter.

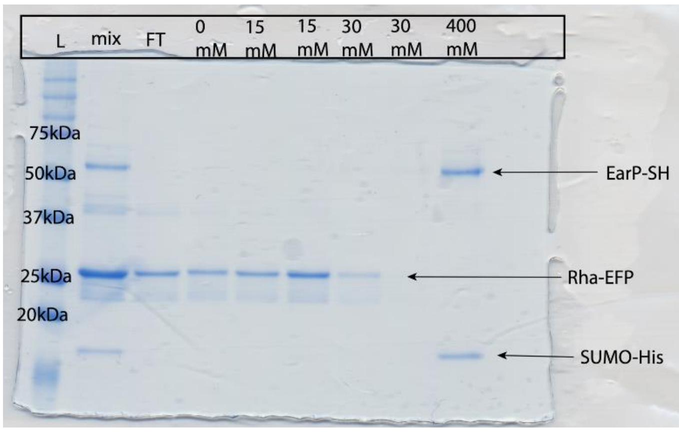

Figure S11. SDS-PAGE of Rha-EFP purification fractions. L=protein ladder, mix = crude reaction mixture, FT=flow-through from the Ni-NTA column. Concentration of imidazole in the fractions is indicated directly above the other lanes. The location of bands belonging to EarP-SH, Rha-EFP and SUMO-His is indicated

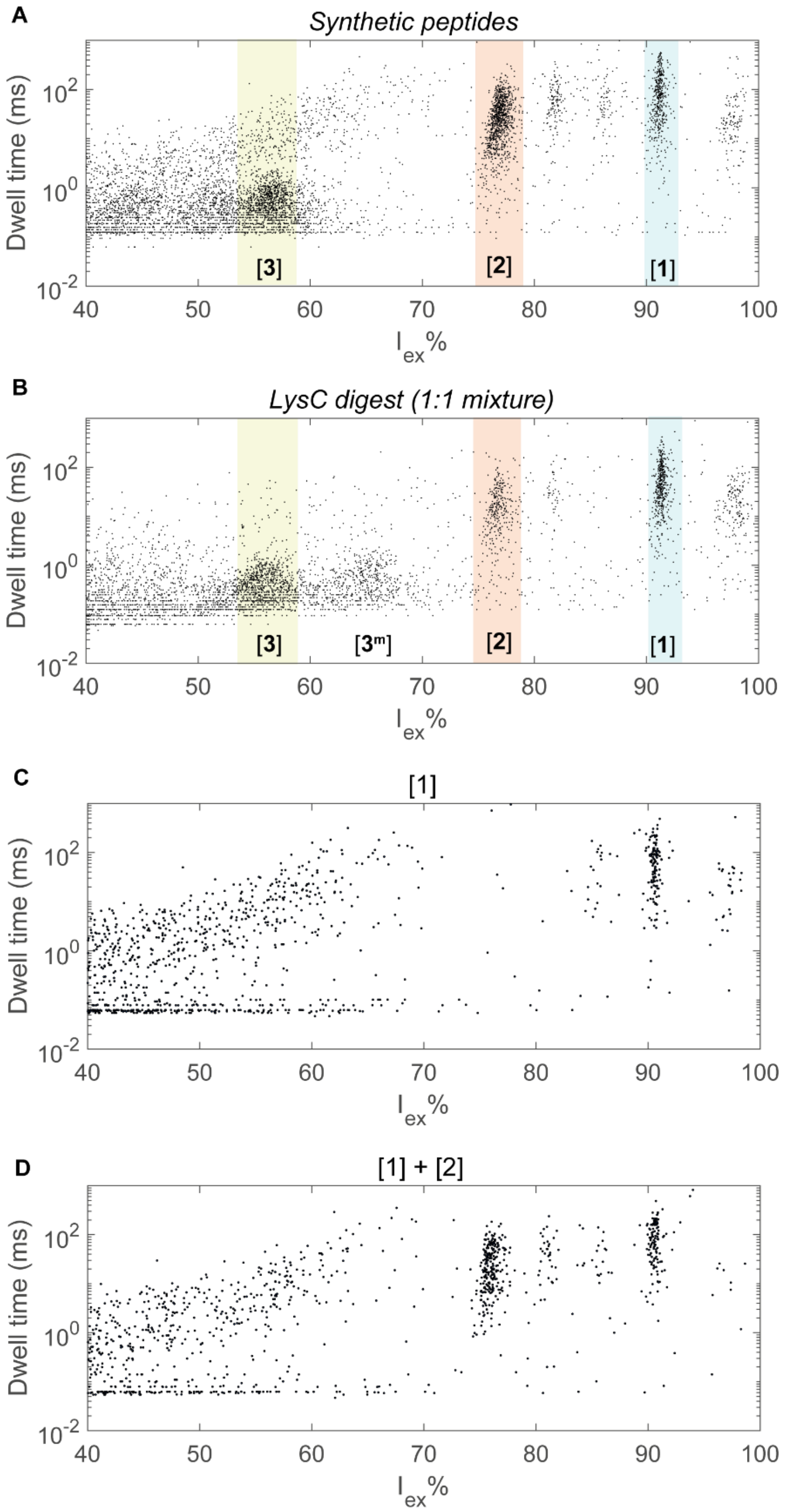

Figure S12: Nanopore spectrum of synthetic EF-P peptides. (A) The four peptides resulting from the complete digestion of EF-P by LysC were added to the nanopore. The location of peptide clusters from peptides **[1]**, **[2]** and **[3]** are highlighted in blue, red and yellow respectively, peptide **[4]** was not detected. (B) Nanopore spectrum of LysC-digested EF-P (a 1:1 (m:m) mixture of EF-P and RhaEF-P). (C) Nanopore spectrum after the addition of peptide **[1]** and (D) nanopore spectrum of a mixture of peptide **[1]** and **[2]**. Measurements in 3M LiCl, buffered to pH 3.8, at -50mV applied voltage. Data was recorded with a 50 kHz sampling frequency and a 10 kHz Bessel filter.

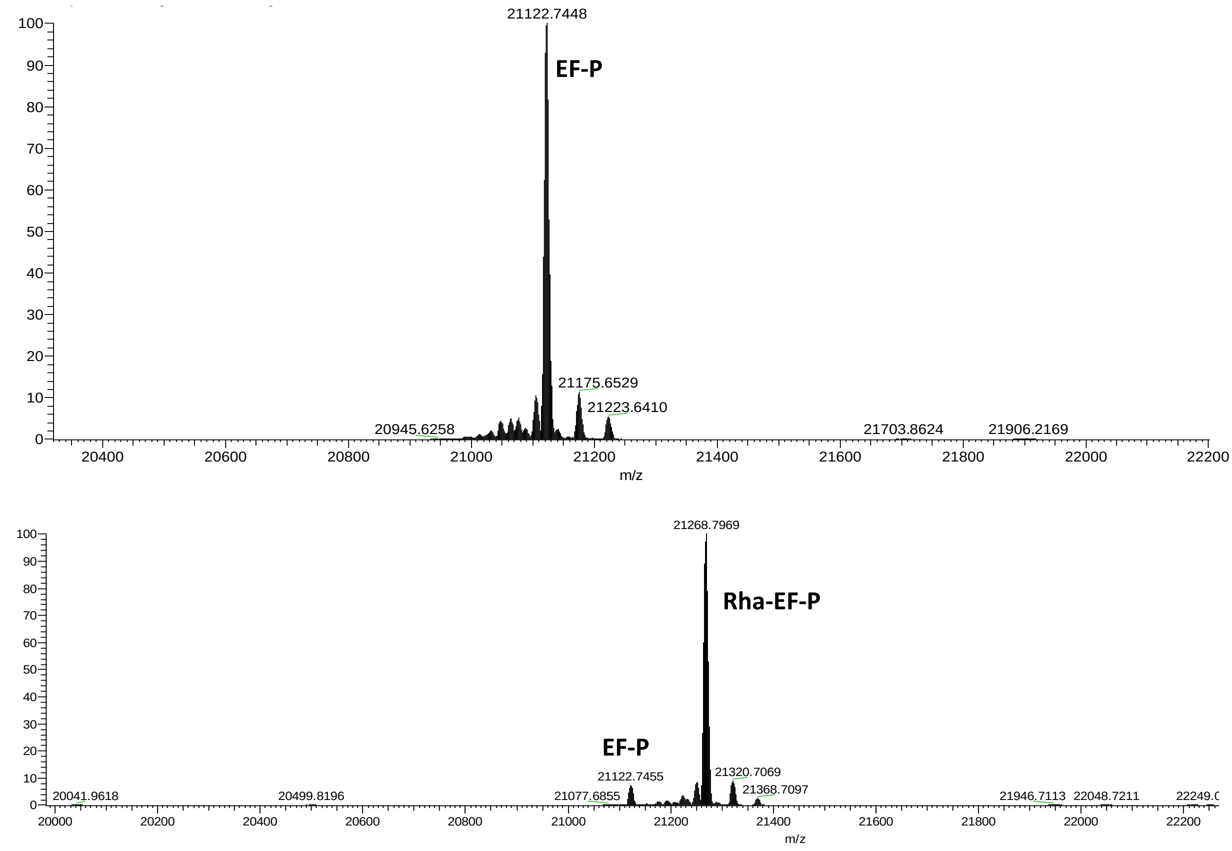

Figure S13: Deconvoluted intact protein mass spectrum of EF-P (top) and rhamnosylated EF-P (bottom). The relative abundance of EF-P in the rhamnosylated sample (bottom) was estimated to be 8%, from which we estimate the yield of rhamnosylation to be (100 – (8/(8+100))) = 92.6%.

Table S1: Event frequency analysis of glycopeptides in 1M KCl

	Total Peptide Concentration (µM)	Measurement time (s)	Events (t_{dwell} >0.1ms)	Event Frequency (Events s^{-1} μM^{-1})
$FraC^{G13F}$ Pore 1	7.5	500	18393	4.90
$FraC^{G13F}$ Pore 2	15	500	23862	3.18
$FraC^{G13F}$ Pore 3	7.5	500	13358	3.56
$FraC^{Wt}$ Pore 1	30	490	603	0.041
$FraC^{Wt}$ Pore 2	30	426	265	0.023

A 1:1:1 equimolar mixture of 9mer_unmod:9mer_1Glc:9mer_2Glc was measured at -50 mV applied voltage in 1M KCl with 50 mM Citric acid, buffered to pH 3.8 using bistris propane. The event frequency was measured by counting the events with a t_{dwell} of at least 100 ms and dividing the number of events by the product of measurement time and total peptide concentration.

Table S2: Detected EF-P peptides in the MS measurement of LysC-digested EF-P.

Peptide		-10lgP	Mass	m/z	RT	Area EF-P	PTM
NLLTGAGTETVFK	[**1**]	90.21	1349.719	675.8667	49.85	7.28E+08	
TAQEFRAGQVANINGAPWVIQK		91.85	2397.255	1199.639	48.74	3.24E+08	
LEPIILDRK	[**2**]	54.03	1095.665	548.8386	34.59	1.45E+08	
SGRNAAVVK	[**3**]	47.96	900.5141	451.2638	1.23	8.91E+06	
SGRNAAVVKMK		52.12	1159.65	387.5571	7.97	1.54E+06	
N(+.98)LLTGAGTETVFK	[**1**]	48.55	1350.703	676.37	50.76	6.50E+05	Deamidation (NQ)
SGRNAAVVKM(+15.99)K		55.9	1175.644	392.8885	3.94	6.03E+05	Oxidation (M)
VISVELPTTIVRQIAYTEPAVRGDTSGK		26.88	2999.629	750.9147	63.31	4.43E+05	
TAQEFRAGQVANIN(+.98)GAPWVIQK		49.23	2398.239	800.4242	59.91	3.52E+04	Deamidation (NQ)
TAQEFRAGQVAN(+.98)INGAPWVIQK		42.38	2398.239	800.4264	64.36	3.29E+04	Deamidation (NQ)
TAQ(+.98)EFRAGQ(+.98)VANINGAPWVIQK		42.11	2399.223	800.762	57.12	1.94E+04	Deamidation (NQ)
TAQEFRAGQVAN(+.98)IN(+.98)GAPWVIQK		36.24	2399.223	800.7611	57.81	8.19E+03	Deamidation (NQ)
TAQEFRAGQ(+.98)VANINGAPWVIQK		47.47	2398.239	800.4248	72.95	5.69E+03	Deamidation (NQ)
NLLTGAGTETVFKADDK		70.91	1778.905	890.4593	39.98	0	

The -10lgP score relates to the probability of detection and the peak area (Area EF-P) relates to the concentration of the peptides in the sample. The second column shows the peptide numbers according to Table 2.

Table S3: Detected EF-P peptides in the MS measurement of LysC-digested rhamnosylated EF-P.

Peptide		-10lgP	Mass	m/z	RT	Area Rham EF-P	PTM
NLLTGAGTETVFK	[**1**]	106.74	1349.72	675.87	47.24	9.08E+08	
TAQEFRAGQVANINGAPWVIQK		132.12	2397.26	1199.64	47.73	4.56E+08	
LEPIILDRK	[**2**]	62.35	1095.67	548.84	35.82	2.13E+08	
SGR(+146.06)NAAVVK	[**3m**]	48.59	1046.57	349.86	1.56	3.73E+06	Rhamnose (R)
SGR(+146.06)NAAVVKMK		52.56	1305.71	436.24	8.64	3.02E+06	Rhamnose (R)
SGR(+146.06)N(+.98)AAVVK	[**3m**]	18.54	1047.56	524.79	7.41	1.65E+06	Rhamnose (R)
N(+.98)LLTGAGTETVFK		52.05	1350.70	676.37	51.34	1.41E+06	Deamidation (NQ)
SGR(+146.06)NAAVVKM(+15.99)K		46.13	1321.70	441.58	7.22	1.19E+06	Rhamnose (R)
VISVELPTTIVRQIAYTEPAVRGDTSGK		30.08	2999.63	750.92	63.26	8.71E+04	
SGRNAAVVK	[**3**]	33.18	900.51	451.26	7.37	3.60E+04	
TAQEFRAGQVAN(+.98)IN(+.98)GAPWVIQK		62.7	2399.22	800.76	71.41	2.89E+04	Deamidation (NQ)
TAQEFRAGQVANIN(+.98)GAPWVIQK		55.07	2398.24	800.43	67.45	1.71E+04	Deamidation (NQ)
SGRNAAVVKM(+15.99)K		61.46	1175.64	392.89	7.22	2.50E+03	Oxidation (M)
NLLTGAGTETVFKADDK		99.44	1778.91	890.47	40.01	0	

The -10lgP score relates to the probability of detection and the peak area (Area Rham EF-P) relates to the concentration of the peptides in the sample. The second column shows the peptide numbers according to Table 2.

Solid-phase peptide synthesis of the 9mer peptide

Peptides were synthesized on a 0.1 mmol scale with a Microwave-assisted Peptide Synthesizer (CEM Liberty Blue peptide Synthesizer): Rink Amide AM resin (146 mg, 100 µmol, 0.684 mmol/g) was swollen in 10 mL of DMF 5 min, drained, and then treated with 20 vol.% piperidine in DMF (10 mL) for 65 seconds at 90°C, drained and washed with DMF (3 x 5 mL). The resin was then treated with a solution of Fmoc-Xaa-OH (0.2 mol/L, 2.5 mL, 5 eq), DIC (1 mol/L, 1 mL, 10 eq) and Oxyma (1 mol/L, 0.5 mL, 5 eq) in DMF (4 mL) at 76°C for 15 s before the temperature was increased to 90°C for an additional 110 s before being drained. The resin was washed with DMF (3 x 5 mL) followed by Fmoc deprotection, 20 vol.% piperidine in DMF (10 mL) for 65 seconds at 90°C, drained and washed with DMF (3 x 5 mL). The resin was treated again with the same amount of the next Fmoc-Xaa-OH, DIC and Oxyma in DMF (4 mL) at 76°C for 15 s before the temperature was increased to 90°C for an additional 110 s before being drained. After coupling and Fmoc deprotection of the final amino acid the N-terminal was acetylated using acetic anhydride/DiPEA (v/v = 1:1.7) in 5 ml DMF for 120 seconds at 60 °C. The peptide was cleaved from the resin using a mixture of TFA/H_2O/TIPS (95:2.5:2.5) while shaking for 2 hours at room temperature. The resin beads were filtered over cotton and precipitated into a mixture of cold MTBE:hexanes (1:1). The precipitate was centrifuged at 4000 rpm for 5 min, washed once more with MTBE:hexanes and centrifuged at 4000 rpm. The crude peptide was lyophilized from ^{t}BuOH:H_2O (1:1) and analyzed by LC-MS and purified by preparative HPLC (0-100%, water-acetonitrile with 0.1% TFA) affording pure peptides as a white solid in 49% (ANVTLNTAG) and 45% (ANVTLNTTG) yield.

ANVTLNTAG (901 Da)

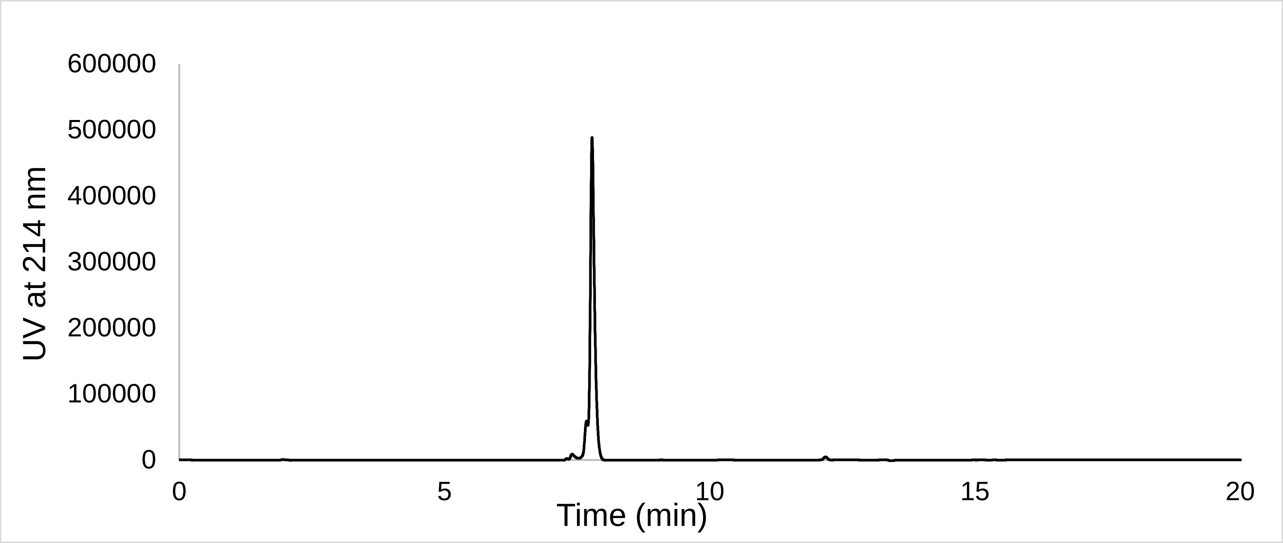

ANVTLNTTG (931 Da)

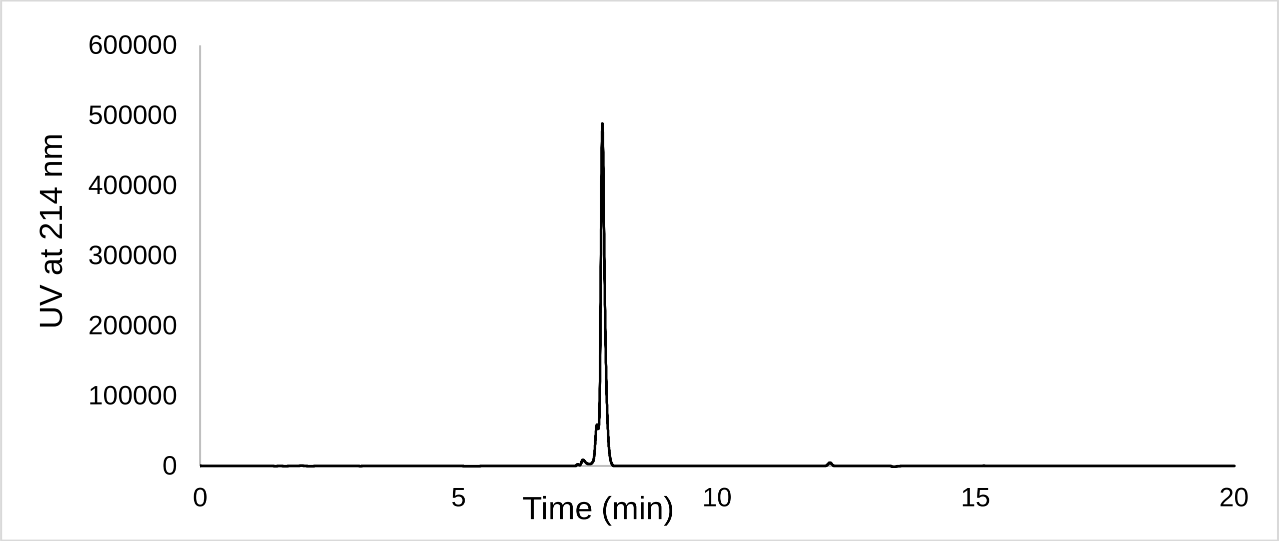

Figure S14: Analytical HPLC traces of unmodified 9mers (ANVTLNTAG (top) and ANVTLNTTG (bottom)),

References

(1) Yakovlieva, L.; Wood, T. M.; Kemmink, J.; Kotsogianni, I.; Koller, F.; Lassak, J.; Martin, N. I.; Walvoort, M. T. C. A β-Hairpin Epitope as Novel Structural Requirement for Protein Arginine Rhamnosylation. *Chem. Sci.* **2021**. https://doi.org/10.1039/D0SC05823H.

(2) Yakovlieva, L.; Ramírez-Palacios, C.; Marrink, S. J.; Walvoort, M. T. C. Semiprocessive Hyperglycosylation of Adhesin by Bacterial Protein N - Glycosyltransferases. *ACS Chem. Biol.* **2021**, *16* (1), 165–175. https://doi.org/10.1021/acschembio.0c00848.

(3) Lucas, F. L. R.; Versloot, R. C. A.; Yakovlieva, L.; Walvoort, M. T. C.; Maglia, G. Protein Identification by Nanopore Peptide Profiling. *Nat. Commun.* **2021**, *12* (1), 5795. https://doi.org/10.1038/s41467-021-26046-9.
